# Treatment of Adolescents with Concurrent Substance Use Disorder and Attention-Deficit/Hyperactivity Disorder: A Systematic Review

**DOI:** 10.3390/jcm10173908

**Published:** 2021-08-30

**Authors:** Heval Özgen, Renske Spijkerman, Moritz Noack, Martin Holtmann, Arnt Schellekens, Søren Dalsgaard, Wim van den Brink, Vincent Hendriks

**Affiliations:** 1 Parnassia Addiction Research Centre (PARC), Parnassia Psychiatric Institute, 2512 HN The Hague, The Netherlands; renske.spijkerman@brijder.nl (R.S.); Vincent.Hendriks@brijder.nl (V.H.); 2 Leiden University Medical Center, 2333 ZA Leiden, The Netherlands; 3 Department of Child and Adolescent Psychiatry, LWL-University Hospital, Hamm, Ruhr-University Bochum, 44801 Bochum, Germany; moritz.noack@lwl.org (M.N.); martin.holtmann@lwl.org (M.H.); 4 Department of Psychiatry, Radboud University Medical Center, Geert Grooteplein Zuid 10, 6525 GA Nijmegen, The Netherlands; Arnt.Schellekens@radboudumc.nl; 5 Donders Institute for Brain Cognition and Behavior, Radboud University Nijmegen, 6525 AJ Nijmegen, The Netherlands; 6 International Collaboration on ADHD and Substance Abuse (ICASA) Foundation, 6500 HE Nijmegen, The Netherlands; w.vandenbrink@amsterdamumc.nl; 7 Department of Economics and Business Economics, Aarhus University, DK-8210 Aarhus, Denmark; sdalsgaard@econ.au.dk; 8 Amsterdam University Medical Centers, Location Academic Medical Center, 1106 AZ Amsterdam, The Netherlands

**Keywords:** review, ADHD, SUD, comorbidity, adolescents, treatment

## Abstract

Childhood attention-deficit/hyperactivity disorder (ADHD) is a risk factor for the development of substance abuse and substance use disorders (SUD) in adolescence and (early) adulthood. ADHD and SUD also frequently co-occur in treatment-seeking adolescents, which complicates diagnosis and treatment, and is associated with poor treatment outcomes. In this study, we provide a systematic review of controlled studies on the effectiveness of pharmacological, psychosocial, and complementary treatments of ADHD in adolescents with and without comorbid SUD. In addition, we review the longitudinal association between pharmacotherapy for childhood ADHD and the development of SUD in adolescence and early adulthood. We conducted a systematic review of the research literature published since 2000 using Medline, PsycINFO, and the Cochrane Database of Systematic Reviews databases to select randomized clinical trials, observational studies, and meta-analyses. The quality of the evidence from each study was rated using the SIGN grading system. Based on the limited evidence available, strong clinical recommendations are not justified, but provisionally, we conclude that stimulant treatment in children with ADHD may prevent the development of SUD in adolescence or young adulthood, that high-dose stimulant treatment could be an effective treatment for adolescents with ADHD and SUD comorbidity, that cognitive behavior therapy might have a small beneficial effect in these patients, and that alternative treatments are probably not effective. More studies are needed to draw definitive conclusions that will allow for strong clinical recommendations.

## 1. Introduction

Childhood attention-deficit/hyperactivity disorder (ADHD) is a risk factor for early substance use initiation and the development of substance use disorders (SUD) in adolescence and (early) adulthood [1,2]. Although we know little about the prevalence of co-occurring ADHD and SUD among adolescents in the general population [3,4], researchers have found high comorbidity rates among adolescents in mental health and substance abuse treatment [5,6]. For example, a meta-analysis on pooled data of nearly 4000 adolescents in substance abuse treatment revealed that 24% of these adolescents were also diagnosed with ADHD [4].

Studies suggest that the co-occurrence of ADHD and SUD can be partly explained by common vulnerability factors, including genetic predispositions [7,8,9] and (associated) dysfunctions in the inhibitory and reward system in the brain [10,11]. In addition, symptoms of ADHD and their consequences may increase the risk of addiction problems, particularly in those with co-occurring oppositional defiant disorder and conduct disorder [1,12]. The reverse relationship—in which substance use results in ADHD—is unlikely, given that ADHD generally develops before initial alcohol or drug use [13,14].

ADHD is found to be more common in males than in females by ratios ranging between 3:1 to 1.5:1 in population-based studies, and ratios ranging between 5:1 and 9:1 in clinical samples. However, an increasing number of studies suggest that females with ADHD face more severe outcomes and more often experience delayed and insufficient treatments than males with ADHD. Sex differences have been reported in the onset and/or type of symptoms, medication use, and quality of life [15,16].

Differences in SUD prevalence rates between males and females are becoming smaller worldwide, with a substantial variation within cultures, with higher rates in cultures where men have better access to substances relative to women. These sex differences in ADHD and SUD have been shown to arise from differences in genetic, hormonal, socioeconomic, environmental, and psychosocial influences; gender bias; and differences in the access to specialty care [17]. Importantly, it seems that there are no sex differences in the prevalence of comorbid ADHD in SUD patients [4], whereas there seem to be slightly more female than male ADHD patients with comorbid SUD [15].

Existing guidelines on ADHD pay relatively little attention to adolescence as a distinctive developmental phase, even though important hormonal, physical, neurobiological, and psychosocial changes take place during this period. These alterations are likely to affect the course of, as well as adherence and response to, ADHD treatment. Moreover, most guidelines pay little or no attention to adolescents with concurrent ADHD and SUD as a distinctive subgroup, and only provide some general advice to screen adolescents with ADHD for symptoms of substance misuse and SUD, to use medications with little or no abuse potential, and to be alert for signs of misuse or diversion of ADHD medication in this group (in countries such as the Netherlands [18], Australia [19], Scotland [20], Germany [21], Canada [22], the UK [23], and the USA [24]). Moreover, recommendations are directed only towards medical practitioners that already have experience in the treatment of ADHD and SUD, and fail to provide guidance to general practitioners, general child psychiatrists and psychologists, or general addiction physicians. All guidelines reflect that the evidence-base pertaining to the treatment of adolescents with concurrent ADHD and SUD is small, and that there is a paucity of research on this comorbidity. Hence, the (few) recommendations for treatment provided on concurrent ADHD and SUD in the guidelines are largely practice-based.

There are several effective treatments for adolescents with ADHD and—although less well studied—for those with SUD. However, the co-occurrence of ADHD and SUD complicates treatment of both disorders and is likely to negatively affect treatment outcomes [25,26,27]. Nevertheless, those developing treatment protocols for the different disorders pay little attention to “the other problem”, and we have no international guidelines for the diagnosis and treatment of their comorbidity in adolescents. In addition, ADHD often remains unrecognized—and hence, untreated—in youth with substance use disorders, and the same is true for the lack of detection of SUD among ADHD patients in youth mental health care [28].

Here, we provide a systematic review of controlled studies on the effectiveness of pharmacological, psychosocial, and complementary treatments of ADHD in adolescents with concurrent ADHD and SUD. As we knew in advance that the literature on the treatment of these comorbid adolescents is limited, controlled studies pertaining to the treatment of adolescents with ADHD but without SUD are also included. Moreover, the inclusion of studies on adolescent ADHD patients with and without SUD will allow us to explore the question of whether poor treatment outcomes in adolescent ADHD patients with concurrent SUD may be related to SUD comorbidity or to lower efficacy of ADHD treatments during the adolescent phase of life. In contrast to most studies on ADHD in youths, we only include studies that exclusively focus on adolescents or report separate outcomes for children and adolescents. We also review the longitudinal association between (stimulant) pharmacotherapy for childhood ADHD and the development of SUD in adolescence and early adulthood.

## 2. Methods

Systematic literature searches were conducted in Medline, PsycINFO, and the Cochrane Database of Systematic Reviews, using the following inclusion criteria:

(a) Studies were original studies published between 2000 and 2021 (11 April 2021) in peer-reviewed, English-language journals.
(b) Studies evaluated the effectiveness of treatment of ADHD in adolescents (12–20 years old) with or without concurrent SUD.
(c) Study treatments involved a pharmacological, psychosocial, or complementary (e.g., dietary) intervention targeted at ADHD.
(d) Diagnoses of ADHD and SUD were based on the DSM-IV/-5 [29] or ICD-10/-11 criteria [30].
(e) Studies were randomized controlled trials (RCTs), controlled clinical trials, randomized cross-over studies, or relevant meta-analyses.
(f) Outcome measures included validated rating scales for ADHD, and—in studies involving patients with comorbid SUD—a quantitative measure of consumption of substances (e.g., days/frequency of use/abstinence).
(g) Studies had to include a minimum of 10 (ADHD plus SUD) or a minimum of 20 (ADHD without SUD) adolescent patients per treatment condition.
(h) Studies in mixed samples of children and adolescents, or adolescents and adults had to meet inclusion criterion.
(i) Studies in mixed samples of children and adolescents, or adolescents and adults had to have separate outcomes analyzed and reported for the adolescent subgroup.

Next, we conducted an additional manual search of the reference sections in the selected papers in order to identify relevant articles, reports, or books that were missed in the systematic searches. Study authors H.Ö., R.S., and V.H. reviewed the titles and abstracts of all identified studies, retrieved and read the full-text manuscripts of those that seemed to meet the inclusion criteria, and made a final decision as to the eligibility of each manuscript, based on consensus. The key characteristics and findings of the included studies were recorded, and risk of bias of each study was assessed, using the updated risk-of-bias tool of the Cochrane Collaboration (RoB-2) [31]. The quality of the evidence from each study (i.e., considering the study design and its methodological quality) was rated using the SIGN grading system [32] (see Table S1 in Supplementary Materials). We followed the Prisma guidelines (see Supplementary Materials Figure S1).

## 3. Results

### 3.1. Literature Search

**Figure 1:**
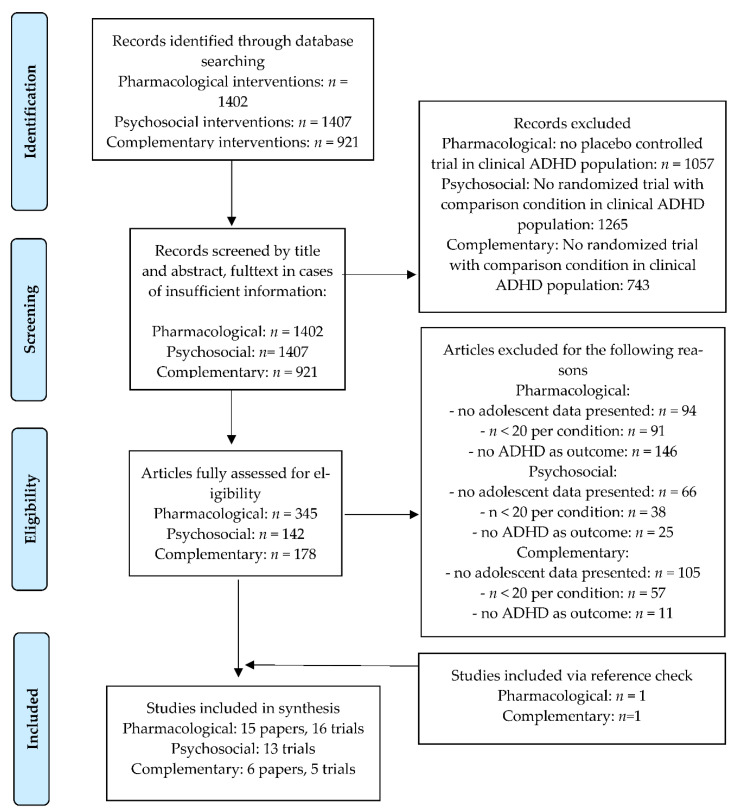
Flowchart.

Outcomes of the literature search are presented in the flowchart in Figure 1. The applied search terms are presented in Table S2 in the Supplementary Materials. Initial database searching resulted in 1402, 1407, and 921 unique records for pharmacological, psychosocial, and complementary interventions, respectively. Based on the first screening, many studies were excluded due to the lack of a controlled design and/or not addressing the effectiveness of an ADHD-focused intervention. Based on a thorough assessment of the remaining articles, we selected 15 papers that included 16 trials for the synthesis on pharmacotherapy, 13 papers with 13 trials on the effectiveness of psychosocial interventions, and 6 papers including 5 trials on the effectiveness of complementary interventions. The main reasons for excluding articles from the final selection were lack of separate outcome data for adolescents, small sample size, and no reporting of an ADHD outcome measure.

### 3.2. Pharmacological Interventions

We identified 16 RCTs on pharmacological treatment that met the selection criteria of our literature search: four placebo-controlled randomized trials in adolescents with concurrent ADHD and SUD (458 enrolled patients; Table 1), and 12 placebo-controlled randomized trials in adolescents with ADHD but without SUD comorbidity (2675 patients; Table 1), with three of them comparing two active medications with placebo [33,34]. In addition, we found one systematic review with a meta-analysis pertaining to the efficacy and safety of the pharmacological treatment of patients with concurrent ADHD and SUD [35].

In their meta-analysis of randomized placebo-controlled trials, Cunill et al. (2014) included four studies on adolescents and nine studies on adults with concurrent ADHD and SUD (1271 patients) using the following ADHD medications: methylphenidate (*n* = 8), atomoxetine (*n* = 3), pemoline (*n* = 1), bupropion (*n* = 1), and lisdexamphetamine (*n* = 1). The mean pooled effect size on ADHD symptoms from all of the studies amounted to an odds-ratio (OR) of 1.93 (small effect). Compared with placebo, methylphenidate (OR = 2.02) and atomoxetine (OR = 1.71) significantly reduced ADHD symptoms, but the other medications did not [35]. None of the medications were effective in increasing abstinence from substances (OR = 1.09). Age of the patients (in years) did not moderate the effect on ADHD symptoms. Unfortunately, this meta-analysis did not distinguish between adolescent and adult patients, and did not consider the heterogeneity within and between samples nor the methodological differences between the studies and the variations in dosing. The four trials on adolescents with ADHD and SUD in the meta-analysis of Cunill et al. (2014) [35] are part of our selection for the present systematic review.

#### 3.2.1. Adolescents with Concurrent ADHD and SUD

Two of the four studies in adolescents with ADHD and SUD involved methylphenidate (MPH) [36,37], one study involved pemoline [38], and one study involved atomoxetine [39] (Table 1). From these, one study required a minimum level of ADHD symptom severity to be included [39], and none of the studies required adolescents to be abstinent at the start of the study treatment.

Riggs et al. (2011) investigated OROS-MPH vs. placebo in a 16-week RCT in 303 adolescents with ADHD and mixed (non-nicotine and non-opioid) SUD, with both treatment groups concurrently receiving once-weekly combined cognitive behavioral therapy (CBT) and motivational interviewing (MI) for SUD. On the primary outcome measures, they found significant baseline to endpoint reductions in self-reported ADHD symptoms and self-reported number of substance use days in both treatment groups, but no between-group difference on either measure. However, some secondary outcomes, including parent-reported ADHD symptoms at week 8 (*p* = 0.02) and week 16 (*p* < 0.001) and the number of negative urine drug screens (*p* = 0.05) favored OROS-MPH, leading the authors to conclude that “(...) further consideration of potential reasons for failed efficacy on the primary outcome measure is warranted (...)” [37] (p. 911).

Szobot et al. (2008) conducted a 6-week randomized cross-over study in 16 male adolescents with ADHD and a comorbid cannabis or cocaine use disorder. They found MPH-SODAS to be more effective than placebo at improving ADHD symptoms and global functioning, but not at reducing substance use [36].

Riggs et al. (2004) compared pemoline with placebo in a 12-week RCT of 69 adolescents with ADHD and comorbid mixed (non-nicotine) SUD and conduct disorder (CD), and the results showed a significant between-group effect in favor of pemoline in terms of the percentage of adolescents with clinician-rated “much improved” or “very much improved” ADHD symptom severity at the study endpoint (*p* = 0.05), but no effect on parent-rated ADHD symptom severity (*p* = 0.13), substance use (adolescent-report: *p* = 0.80; urinalysis: *p* = 0.33), and CD symptoms (*p* = 0.44). Due to hepatotoxicity concerns, pemoline was withdrawn from the market in 2005 [38].

Lastly, Thurstone et al. (2010) conducted a 12-week RCT comparing atomoxetine max. 100 mg/day and placebo in 70 adolescents with ADHD and mixed (non-nicotine) SUD who concurrently received once-weekly CBT/MI for SUD, and found no between-group difference in improvement for neither ADHD or substance use, both based on self-report and urinalysis [39].

In all four trials, pharmacological treatment was well tolerated. Adverse events (AEs) and/or treatment emergent AEs (TEAEs) were generally more prevalent in patients in the active medication groups, but these were mostly mild in intensity and transient. In the trial of pemoline, no elevation of liver enzyme levels was observed. Study-related serious adverse events (SAEs) in the active medication groups were absent or rare (≤1 SAE) in all studies, with no excess of SAEs in any active medication group compared with placebo. Adverse pharmacological interactions between the study medication and the adolescent’s substance use at the same day were only reported in the trial of Riggs et al. (2011) [37], and only by four (2.8%) and three (2.1%) of the patients taking OROS-MPH and placebo, respectively. In the three trials that investigated stimulants, no indication was found that stimulant medication led to SUD deterioration.

Planned treatment duration in these trials ranged from 6–16 weeks (1 of 4 studies >12 weeks), and no subsequent extension studies were conducted to investigate the long-term effects of ADHD medication in the study samples. Hence, pertaining to adolescents with comorbid ADHD and SUD, only data on the short-term effects of ADHD medication are available to date. We assessed the risk of bias as being low in the studies of Riggs (2011) and Thurstone (2010), and as high in the studies of Szobot (2008) and Riggs (2004), mainly due to missing outcome data (Table 2).

To conclude, the evidence base on pharmacological ADHD treatment in adolescents with concurrent ADHD and SUD is limited, with less than 500 patients included across four controlled trials of a short duration, none of which showed a robust treatment effect on either ADHD or SUD.

#### 3.2.2. Adolescents with ADHD but without SUD Comorbidity

Most trials on the efficacy of pharmacological ADHD treatment in youth have been conducted in mixed samples of children and adolescents at an age group typically ranging from 5 to 18 years, without separate analysis or reporting of outcomes in the adolescent subgroup. Our selected literature included a meta-analysis by Cerrillo-Urbina et al. (2018) of 15 RCTs comparing stimulant and non-stimulant medications with placebo in children and adolescents with ADHD [48]. Only four of these, 15 trials focused on adolescents only. The pooled standardized mean effect size (SMD; equal to Cohen’s d) of the three adolescent studies of stimulant medications on ADHD symptoms was 0.66, with substantial heterogeneity (I2 = 77%; *p* = 0.01), and the SMD of the one non-stimulant medication was 0.52. We included all four adolescent studies from this meta-analysis in our review below.

In our literature search, we found 12 trials in adolescents with ADHD without SUD comorbidity that met our selection criteria. Five of these involved MPH [27,33,34,40], one study involved lisdexamfetamine (LDX) [42], one study involved mixed amphetamine salts extended release (MAS-XR) [41], two studies were about pemoline (one of which compared both pemoline and MPH with placebo) [33,43], one study used atomoxetine [44], and three studies tested the effect of guanfacine [45,46,47] (Table 1). From these, six studies required a minimum ADHD symptom severity to be included [34,42,44,46,47], six studies excluded patients with a history of non-response to the study medication or to stimulants prior to study entry [27,34,40,41,42], and one study required a favorable response to the study medication in the open-label titration phase to be included in the double-blind study phase [40].

With the exception of one pemoline study [33] and the adolescent subgroups in two guanfacine studies [45,46], all studies reported significant improvements in ADHD symptoms in the active medication compared with the placebo groups, with moderate to large effect sizes of Cohen’s d = 0.53–1.33 for MPH, d = 0.80–1.23 for LDX, d = 0.80 for MAS-XR, d = 2.05 for pemoline, d = 0.99 for atomoxetine, and d = 0.52 for guanfacine (Table 1).

As in adolescents with ADHD and SUD, AEs and/or TEAEs generally occurred more often in patients in the active medication groups. The most reported AEs in the studies that investigated stimulants were decreased appetite and weight loss, headache, irritability, insomnia, and abdominal pain. Atomoxetine treatment was associated with decreased appetite and weight loss, nausea, dizziness, and diarrhea, and guanfacine treatment was associated with insomnia, sedation, fatigue, and abdominal pain. In all studies, most AEs were mild to moderate in intensity. Changes in ECG parameters, pulse rate, and blood pressure were more prevalent in nearly all active medication groups, but were judged as not clinically meaningful in all studies. The SAEs in the active medication groups were absent or rare (≤1 SAE) in all studies.

Planned treatment duration of the double-blind phase in these studies ranged from 2–13 weeks (1 of 12 studies >12 weeks). Open-label extension studies to the previous trials in adolescents have been conducted for MPH [27,49], LDX [40], MAS-XR [40,41], and atomoxetine [44], with follow-up periods ranging from 2–12 months. In these follow-up studies, efficacy was maintained, and side-effects and tolerability were consistent with those found in the antecedent, controlled studies. However, we cannot draw firm conclusions about the long-term safety and efficacy of these medications from these studies, due to their open-label character and given that only a selection of subjects of the antecedent studies participated in these follow-up studies.

We rated the risk of bias as low in the randomized studies of Findling et al. (2011) [42], Newcorn et al. (2017b) [34], and Spencer et al. (2006) [25], and as high in all other studies (Table 2).

To conclude, 12 randomized trials with a total of more than 2600 adolescent patients with ADHD without SUD comorbidity show robust, moderate-to-large effects on ADHD symptoms of both stimulant and non-stimulant medications compared with placebo.

### 3.3. Childhood ADHD and Later SUD

We also reviewed the literature on childhood ADHD as a risk factor for developing SUD in adolescence and early adulthood, as well as the literature on the effect of pharmacological ADHD treatment for children on the development of later SUD.

#### 3.3.1. Childhood ADHD and the Risk of Later SUD

To evaluate the association between childhood ADHD and the risk of developing a SUD during adolescence or early adulthood, we reviewed four (partly overlapping) meta-analyses of longitudinal studies that compared children with and without ADHD [1,2,50,51], and two large-scale (*n* = 547 to 1017) prospective cohort studies [51,52].

In all four meta-analyses, childhood ADHD was associated with an increased risk of SUD in adolescence or early adulthood, compared with non-ADHD controls, with mean odds-ratios (OR) ranging from 1.34 to 3.48 (small-to-moderate association) for different types of SUD. In the prospective case-control study of Groenman et al. (2013) [52], childhood ADHD was associated with an increased risk of developing SUD in adolescence, and with a hazard ratio (HR) of 1.77 compared with the healthy controls. Risk of SUD was the highest in children with concurrent ADHD and CD, but children without the CD comorbidity were still at increased risk. In the Multimodal Treatment Study of Children with ADHD (MTA) [53], childhood ADHD was associated with more frequent use of cigarettes and cannabis in young adulthood, but no differences were found for alcohol or illicit drug use.

#### 3.3.2. Stimulant Treatment of Childhood ADHD and the Risk of Later SUD

In the first meta-analysis of six longitudinal studies on the association between (mostly stimulant) pharmacotherapy for childhood ADHD and later risk of SUD in adolescence or early adulthood, Wilens et al. (2003) found a significantly lower risk of later SUD (mean OR = 1.9) in ADHD-children who had received stimulant treatment compared with those who had not [54].

However, in the meta-analysis of 15 longitudinal studies by Humphreys et al. (2013), the studies on ADHD-children with and without stimulant medication showed inconsistent outcomes with positive, neutral, and negative outcomes, but overall, they had a similar risk of developing SUD later in life [55]. Moreover, this review did not consider possible differences in ADHD-severity and comorbid CD between ADHD children that were or were not treated with stimulants.

We also reviewed four prospective cohort studies that were published after Humphreys’ meta-analysis. Molina et al. (2013) compared children in the MTA-cohort with high versus low exposure to stimulant treatment and found no indications for a harmful or beneficial effect of treatment for developing SUD in adolescence [56]. In a prospective follow-up study by Groenman et al. (2013), stimulant treatment of childhood ADHD was associated with a lower risk of later SUD, but not of nicotine dependence, even after controlling for comorbid CD (HR = 1.91) [57]. Dalsgaard et al. (2014) prospectively followed children and adolescents with ADHD and found that those with later vs. earlier stimulant treatment initiation had a higher risk of developing SUD in adulthood (HR = 1.46) [58]. Groenman et al. (2019) distinguished three mutually exclusive subgroups of ADHD-children with distinct stimulant medication trajectories and found that a stimulant treatment profile characterized by an early start, high dose, and long duration was associated with a reduced risk of SUD in adolescence [59].

Lastly, we reviewed two large health care registry studies that investigated the longitudinal association between stimulant ADHD medication and later substance-related events (e.g., death, crime, and emergency department visits). Chang et al. (2014) studied linked national registers that included nearly 39,000 patients with ADHD, and found stimulant ADHD medication to be associated with a 31% lower rate of substance-related events three years later. Moreover, a longer duration of medication was associated with lower rates of events [60]. Quinn et al. (2017) conducted within-individual analyses of registered health care data from nearly three million individuals with ADHD and found that ADHD medication—mostly stimulants—was associated with a 14 to 19% reduction in the odds of an SUD-related emergency department visit two years later, with the largest reduction among adolescents [61].

Taken together, we conclude that (1) childhood ADHD is a serious risk factor for developing SUD in adolescence and early adulthood, (2) studies strongly suggest that stimulant treatment of childhood ADHD does not increase the risk of developing SUD in adolescence, and (3) stimulant treatment of childhood ADHD may have a protective effect on the development of SUD in adolescence and early adulthood. The overall effect size of the reduced risk is probably small, but one of the studies suggests that an early start of stimulant treatment with adequate doses is associated with a moderate to large protective effect. It should be noted, however, that a naturalistic prospective study—which is probably the only suitable and feasible method to study the long-term effects of stimulant treatments in children with ADHD—does not allow for causal inferences and may be affected by unmeasured confounders [59].

### 3.4. Psychosocial Interventions

Psychosocial interventions to treat ADHD include CBT and behavioral therapeutic approaches, motivational interviewing (MI), psychoeducation, parent training, and training to improve planning, organizational skills, social skills, and academic/homework skills. These interventions can be aimed at adolescents themselves or at their social environment (e.g., parents) and effects are often evaluated across different targets, settings, and outcome measures, including ADHD symptoms, social, planning and organizational skills, academic performance, etc.

#### 3.4.1. Adolescents with Concurrent ADHD and SUD

There are no meta-analyses or RCTs on the efficacy of psychosocial treatments in adolescents with concurrent ADHD and SUD.

#### 3.4.2. Adolescents with ADHD but without SUD Comorbidity

Our literature search on psychosocial interventions yielded 13 trials, with 1812 participants (range 46–326) fulfilling our selection criteria (Table 3). Five studies examined psychosocial interventions provided to students with ADHD in a school setting [62,63,64,65,66] and eight studies tested psychosocial interventions in a clinical setting [67,68,69,70,71,72,73,74]. The studies included family-focused parenting interventions or adolescent-focused cognitive behavioral and/or MI-based interventions either with or without a parent component. All interventions targeted ADHD symptoms, as well as social, organization, planning, and academic skills. We rated risk of bias (ROB 2, see Table 4) as high for 10 studies and as having “some concerns” for three of the 13 studies. The main sources of bias were lack of blinding, no independent outcome assessors, no predefined analysis and primary outcome measure, and missing outcome data.

In five of the six trials with a non-active control condition (waiting-list control group), participants in the intervention group showed stronger post-treatment improvements in ADHD-symptoms compared with participants in the control group [62,63,64,65,73,74]. In contrast, seven of the nine studies that included an active control condition (e.g., treatment as usual) did not find any differences in ADHD symptom ratings between the intervention and the control treatment [65,67,68,69,70,71,72]. Limitations of the reviewed studies include the large variation in concomitant medication use and the lack of control of additional psychosocial interventions, which are likely to confound the study outcomes. Furthermore, studies showed great diversity in the setting, content, intensity, and duration (if reported) of treatment.

In sum, randomized trials of psychosocial treatment in adolescents with concurrent ADHD and SUD are non-existent, and the results from psychosocial trials in ADHD adolescents without concurrent SUD are mixed, suggesting that some benefit from treatment when compared with non-active control conditions, but no benefits compared with active control treatments. Between-study heterogeneity was high and the overall study quality was low. Hence, the evidence on psychosocial treatment in ADHD adolescents—with or without concurrent SUD—does not allow for conclusions about which treatments are (most) effective and should be preferred.

### 3.5. Complementary Interventions

The potential benefits of a wide range of complementary interventions have been studied in patients with ADHD, including computerized cognitive training programs (working memory training such as Cogmed), neurofeedback, dietary interventions (e.g., elimination diets, and herbal, mineral, and vitamin supplements), meditation/mindfulness-based therapies, physical exercise, and traditional medicine.

#### 3.5.1. Adolescents with Concurrent ADHD and SUD

There are no meta-analyses or RCTs on the efficacy of complementary interventions in adolescents with concurrent ADHD and SUD.

#### 3.5.2. Adolescents with ADHD but without SUD Comorbidity

Our systematic literature search and an additional investigation of the available meta-analyses on complementary interventions in mixed age groups resulted in five randomized trials that examined the effectiveness of complementary treatment in adolescents with ADHD without concurrent SUD (Table 5). Two trials examined the effects of the Cogmed working memory training in adolescents with ADHD [66,77], one trial reported on the effectiveness of EEG-neurofeedback as an adjuvant therapy to treatment as usual [76], one trial tested the effectiveness of a 12-week regimen with Omega-3/6 fatty acids [78], and one trial examined the potential benefits of a 10-week physical exercise program to reduce ADHD symptoms in adolescents with ADHD [75]. None of the studies showed robust beneficial effects and the study quality was generally low.

We conclude that randomized trials on complementary treatment in adolescents with concurrent ADHD and SUD are absent and that the methodological quality in the few trials in ADHD adolescents without concurrent SUD is insufficient to draw firm conclusions about their efficacy.

## 4. Discussion

Despite the high rate of concurrent ADHD and SUD among adolescents in both addiction and mental health treatment services, the evidence pertaining to the efficacy of treatments for this comorbidity is limited and does not allow for strong recommendations.

Concerning pharmacotherapy, the results of the reviewed randomized trials in adolescents with concurrent ADHD and SUD were equivocal, with contradictory findings between the primary and secondary outcome measures pertaining to ADHD [37,38] and SUD [37], overall negative findings for both ADHD and SUD [39], and a small sample size [36]. Hence, none of the pharmacological trials in this comorbid adolescent population showed a robust between-group effect of treatment on either ADHD or SUD. In contrast, virtually all trials in ADHD adolescents without concurrent SUD showed a significant effect of the medication—MPH, LDX, MAS-XR, GXR, and atomoxetine—on ADHD, with effect sizes in the moderate to large range.

Notably, the overall negative and/or equivocal outcomes in concurrent ADHD and SUD adolescent samples were also found among adults with concurrent ADHD and SUD, except for two trials [79,80] in which a dose much higher than the standard dose of stimulant medication was prescribed (Table S3 in Supplementary Materials). Hence, whereas there is ample evidence to support the efficacy of pharmacological interventions in adolescents with ADHD without concurrent SUD, the evidence of ADHD pharmacotherapy pertaining to both ADHD and substance abuse outcomes in patients with comorbid ADHD and SUD is virtually absent (adolescents) or limited at best (adults).

Several factors may account for or contribute to the discrepancy in the study findings between the concurrent SUD and non-SUD ADHD samples, which include possible differences in (1) patient characteristics other than SUD per se, (2) treatment retention and adherence to the study treatment regimen, (3) medication dose, and (4) the nature of and response to the control or concomitant psychosocial treatment.

Firstly, concerning patient characteristics, the adolescent samples with and without concurrent SUD predominantly included males, and showed similar ADHD symptom severity and a similar ADHD subtype distribution at study entry. However, adolescents with SUD comorbidity were on average nearly two years older than their non-SUD counterparts—which likely reflects the increasing prevalence of SUD with increasing age—and had a high prevalence of concurrent CD, which was mostly absent in the non-SUD samples. However, evidence suggests that age plays a minimal role in moderating the efficacy of stimulant treatment in children, adolescents, and adults with ADHD [35,81], and several studies in children found that ADHD outcomes of pharmacological treatment were similar among those with and without concurrent CD [82,83,84]. In addition, in the trial of Riggs et al. (2011), comorbid CD in adolescents with ADHD and SUD (one-third of the study sample) did not moderate the effect of OROS-MPH vs. placebo on ADHD outcomes [85]. Hence, differences in patient characteristics other than SUD, including age and CD comorbidity, are unlikely to account for the discrepancy in ADHD outcomes between the SUD and non-SUD studies reviewed.

Secondly, medication adherence, if reported, was similarly high (>82%) among adolescents with and without SUD, as was the overall rate of treatment completers in the active medication groups (Table 1).

Thirdly, several authors have suggested that efficacious stimulant treatment may require higher doses in ADHD patients with comorbid SUD [36,86,87], because of decreased brain dopamine function resulting from chronic drug use [10]. As mentioned above, the only two trials with a significant effect on both ADHD and SUD outcomes to date indeed used a much higher than the standard dose of stimulant medication [79,80]. In search for an explanation for these findings, a single photon emission computed tomography (SPECT) study in 24 ADHD patients with (*n* = 8) and without (past) cocaine dependence (*n* = 16) showed a lower striatal dopamine transporter (DAT) occupancy by MPH in cocaine dependent compared with non-dependent ADHD patients after two weeks of MPH treatment. This group difference in DAT occupancy was significantly associated with self-reported impulsivity and craving, but not with the reduction in ADHD symptoms following MPH treatment [88]. However, the sample was small and larger studies are needed to better explain the differences in the effect of stimulant treatment in ADHD patients with and without SUD.

Fourthly, in the reviewed trials, adolescents with and without concurrent SUD showed similar mean ADHD symptom reductions in the active medication groups, but the symptom reductions in the placebo control groups were much larger among those with comorbid SUD [37,39] than in their non-SUD counterparts. A high response to control treatment was also observed in several trials among adults with SUD comorbidity. This suggests that not a lack of medication effect, but rather an inflated placebo response is responsible for the lack of effect on ADHD in the pharmacotherapy trials in patients with concurrent ADHD and SUD. However, it should be noted that control treatment in most of these trials consisted of both placebo and behavioral treatment—CBT/MET for SUD—which was also provided as concurrent treatment in the active medication groups, whereas most control treatments in the non-comorbid samples consisted of placebo only. Hence, given that CBT and MET are effective treatments for SUD and have shown promise in the treatment of ADHD in both adolescents [73] and adults [89] with SUD, CBT/MET, placebo, or both may have caused a ceiling effect, which in turn may have contributed to the failure of pharmacotherapies to separate from the control treatment in the trials on comorbid ADHD and SUD.

Concerning psychosocial treatment, no randomized trials or meta-analyses have been conducted to date in youth with concurrent ADHD and SUD. Although a few trials in ADHD adolescents without concurrent SUD provide some indication of a small beneficial effect, the study quality was too low to draw a firm conclusion about the efficacy of the psychosocial treatment of ADHD in adolescents either with or without concurrent SUD. Given the recommendations in national guidelines that ADHD pharmacotherapy should be embedded in psychosocial treatment, it is obvious that further research is needed to investigate the efficacy of psychosocial treatment in this specific age group. Finally, for complementary interventions, research on adolescents with concurrent ADHD and SUD was absent as well, and the literature on adolescents with ADHD without concurrent SUD was too limited to provide additional clues about treatment efficacy.

## 5. Conclusions

Treatment of adolescents with concurrent ADHD and SUD remains challenging. ADHD is one of the main developmental risk factors for early substance use, misuse, and SUD in adolescence and early adulthood. Early treatment of childhood ADHD (especially with stimulants) may have a small protective effect against the development of SUD in ADHD patients, although the mechanism of such potential protection is unknown and confounds are still possible.

Treatment of adolescents with comorbid ADHD and SUD is still in its infancy, however, pharmacotherapy with higher than standard doses if needed should be explored within the context of a beneficial psychosocial environment and without unnecessary exposure of these adolescents to unpleasant complementary treatment [90]. In addition, we need more research investigating the efficacy of psychosocial and complementary inventions both in ADHD-only patients and in patients with comorbid ADHD and SUD.

## Figures and Tables

**Table 1:** Placebo-controlled studies of pharmacological treatment in adolescents with ADHD and comorbid SUD, and in adolescents without comorbid SUD.

Author, Year	Population	SUD Diagnosis	Study Design and Treatment	Concurrent Treatment	Treatment Completers	Primary Outcome Measurement: ADHD	Primary Outcome Measurement: SUD	Primary Outcome	Level of Evidence (SIGN)
		**Adolescents with ADHD and SUD**							
		Methylphenidate							
Szobot, 2008 [36]	16 adolescents aged 15–21 years (mean 17.4 years; 100% male) with ADHD and cannabis an/or cocaine use disorder (DSM-IV)	Dependence: cannabis (93.8%) or cocaine (43.8%)	6-week, randomized, SB, PC, crossover study: 2 × 3 weeks: MPH-SODAS (0.3–1.2 mg/kg/day) or placebo	No concurrent Tx for ADHD or SUD	87.5% in both treatment conditions combined	Mean change over time on mother-reported SNAP-IV and investigator-rated CGI by treatment condition	Mean change over time in adolescent-reported days of substance use past 1 week by treatment condition	Significant improvement in ADHD symptoms and global functioning (both: *p* ≤ 0.001) in MPH-SODAS condition vs. placebo; no treatment effect on substance use	2 −
Riggs, 2011 [37]	303 adolescents aged 13–18 years (mean 16.5 years; 78.9% male) with ADHD and SUD (DSM-IV)	Dependence: cannabis (66.7%), alcohol (29.7%), cocaine (7.3%), hallucinogen (5%), sedative (2.6%), amphetamine (1.3%), or other (1.2%) Abuse: alcohol (26.4%), cannabis (25.4%), opiate (12.2%), hallucinogen (7.6%), sedative (7.3%), amphetamine (3%), cocaine (2.6%), or other (4%)	16 weeks, RCT, DB, PC; parallel groups: OROS-MPH (max. 72 mg/day, titrated fixed dose) or placebo	16-week individual CBT/MET for SUD	OROS-MPH: 78.1%; placebo: 71.7%	Mean change over time on adolescent-reported ADHD-RS-IV by treatment group	Mean change over time in adolescent-reported days of substance use past 4 weeks (TLFB) by treatment group	No significant difference between both groups in reduction of ADHD symptoms (d = 0.22; ns) and substance use (d = 0.05; ns)	1 +
		Pemoline							
Riggs, 2004 [38]	69 adolescents aged 13–19 years (mean 15.8 years; 84.1% male) with ADHD, SUD, and CD (DSM-IV)	Dependence: alcohol (47.8%) or cannabis (73.9%)	12 weeks, RCT, DB, PC; parallel groups: pemoline (75–112.5 mg/day) or placebo	No concurrent Tx for ADHD, SUD or CD	Pemoline: 54.3%; placebo: 50.0%	% responders on clinician-rated CGI (ADHD-symptoms “much improved” or “very much improved”) at study endpoint and mean change over time on parent-rated CHI by treatment group	Mean change over time in adolescent-reported days of substance use past 30 days (TLFB) and total number of negative urine drug screens by treatment group	Significantly more responders in pemoline group vs. placebo (d = 0.5; *p* = 0.05); no treatment effect on ADHD symptoms on CHI (d = 0.34; ns) and substance use (d = 0.05; ns)	1 −
		Atomoxetine							
Thurstone, 2010 [39]	70 adolescents aged 13–19 years (mean 16.1 years; 78.6% male) with ADHD and SUD (DSM-IV)	Alcohol (28.6%), cannabis (95.7%), cocaine (2.9%), amphetamine (1.4%), or hallucinogen (1.4%)	12 weeks, RCT, DB, PC; parallel groups: atomoxetine (<70 kg: 1.1–1.5 mg/kg/day; ≥70 kg: max. 100 mg/day) or placebo	12-week individual CBT/MET for SUD	Atomoxetine: 91.4%; placebo: 94.3%	Mean change over time on an adolescent-reported DSM-IV checklist by treatment group	Mean change over time in adolescent-reported days of substance use past 4 weeks (TLFB) by treatment group	No significant difference between both groups in reduction of ADHD symptoms (d = 0.10; ns) and substance use (d = 0.35; ns)	1 +
		**Adolescents with ADHD, without SUD**							
		Methylphenidate							
Wilens 2006 [40]	177 adolescents aged 13–18 years (mean 14.6 years; 80.2% male) with ADHD (DSM-IV) without a history of nonresponse to MPH, who responded favorably to OROS-MPH in an open-label titration phase		2 weeks, RCT, DB, PC; parallel groups: OROS-MPH (18–72 mg/day, titrated fixed dose) or placebo	Subjects in behavioral treatment at study enrollment could continue their treatment	OROS-MPH: 81.6%; placebo: 68.9%	Mean change over time on adolescent-reported ADHD-RS-IV by treatment group	NA	Significant improvement in ADHD symptoms in OROS-MPH group vs. placebo (d = 0.56; *p* = 0.001)	1 −
Findling, 2010a [27]	217 adolescents aged 13–17 years (mean 14.6 years; 74.4% male) with ADHD (DSM-IV-TR)		7 weeks, RCT, DB, PC; parallel groups: MTS (10, 15, 20, or 30 mg/day, titrated fixed dose) or placebo	No concurrent Tx	MTS: 65.5%; placebo: 40.3%	Mean change over time on adolescent-reported ADHD-RS-IV by treatment group	NA	Significant improvement in ADHD symptoms in MTS group vs. placebo (d = 1.33; *p* < 0.001)	1 −
Pelham, 2013 [33]	30 adolescents aged 12–17 years (mean 14.1 years; 90% male) with ADHD (DSM-III)		12-week, randomized, DB, PC, conditions cross-over study: IR-MPH (max. 75 mg/day, titrated dose) or pemoline (max. 112.5 mg/day, titrated dose) or placebo in a naturalistic school setting	Not reported	Not reported	Mean change over time on teacher-reported inattention/overactivity subscale of IOWA-CRS by treatment condition	NA	Significant improvement in ADHD symptoms in IR-MPH condition vs. placebo (d = 0.53; *p* < 0.05), but not in pemoline condition vs. placebo (d = 0.21; ns)	2 −
Newcorn 2017a [34]	464 adolescents aged 13–17 years (mean 14.7 years; 66.4% male) with ADHD (DSM-IV-TR)		8 weeks, RCT, DB, PC; parallel groups: OROS-MPH (12–72 mg/day, titrated flexible dose) or LDX (30–70 mg/day, titrated flexible dose) or placebo	Not reported	OROS-MPH: 84.9%; LDX: 83.3%; placebo: 73.1%	Mean change over time on parent-reported ADHD-RS-IV by treatment group	NA	Significant improvement in ADHD symptoms in OROS-MPH (d = 0.97; *p* < 0.0001) and LDX (d = 1.16; *p* < 0.0001) vs. placebo. No significant difference between OROS-MPH and LDX (*p* = 0.07)	1 −
Newcorn 2017b [34]	549 adolescents aged 13–17 years (mean 14.7 years; 66.0% male) with ADHD (DSM-IV-TR)		6 weeks, RCT, DB, PC; parallel groups: OROS-MPH (72 mg/day, titrated fixed dose) or LDX (70 mg/day, titrated fixed dose) or placebo	Not reported	OROS-MPH: 84.5%; LDX: 82.6%; placebo: 88.2%	Mean change over time on parent-reported ADHD-RS-IV by treatment group	NA	Significant improvement in ADHD symptoms in OROS-MPH (d = 0.50; *p* < 0.0001) and LDX (d = 0.82; *p* < 0.0001) vs. placebo. Significant improvement in LDX vs. OROS-MPH (d = 0.33; *p* = 0.0013)	1 +
		(Lis)dexamphetamine/mixed amphetamine salts							
Spencer, 2006 [41]	287 adolescents aged 13–17 years (mean 14.2 years; 65.5% male) with ADHD (DSM-IV-TR)		4 weeks, RCT, DB, PC; parallel groups: MAS-XR (10, 20, 30, 40 mg/day, forced dose titration) or placebo	Not reported	92.8% in all treatment groups combined	Mean change over time on adolescent-reported ADHD-RS-IV by treatment group	NA	Significant improvement in ADHD symptoms in MAS-XR group vs. placebo (d = 0.80; *p* ≤ 0.001)	1 +
Findling, 2011 [42]	314 adolescents aged 13–17 years (mean 14.6 years; 70.3% male) with ADHD (DSM-IV-TR)		4 weeks, RCT, DB, PC; parallel groups: LDX (30, 50, 70 mg/day, forced dose titration) or placebo	Subjects in behavioral treatment at study enrollment could continue their treatment	LDX: 83.4%; placebo: 87.3%	Mean change over time on adolescent-reported ADHD-RS-IV by treatment group	NA	Significant improvement in ADHD-symptoms in LDX groups vs. placebo (30 mg: d = 0.80; 50 mg: d = 1.23; 70 mg: d = 1.09; all: *p* ≤ 0.0056)	1 +
		Pemoline							
Bostic, 2000 [43]	21 adolescents aged 12–17 years (mean 14.1 years; 85.7% male) with ADHD (DSM-IV)		10-week, randomized, DB, PC, crossover study: 2 × 4-week: pemoline (1–3 mg/kg) or placebo	Not reported	71.4% in both treatment groups combined	Mean change over time on adolescent-reported ADHD-RS-IV by treatment condition	NA	Significant improvement in ADHD symptoms in pemoline condition vs. placebo (d = 2.05; *p* = 0.001)	2 −
		Atomoxetine							
Bangs, 2007 [44]	142 adolescents aged 12–18 years (mean 14.5 years; 73.2% male) with ADHD and major depression (DSM-IV)		9 weeks, RCT, DB, PC; parallel groups: atomoxetine (1.2–1.8 mg/kg/day) or placebo	No concurrent Tx	Atomoxetine: 81.9%; placebo: 87.1%	Mean change over time on parent-reported ADHD-RS-IV and CDRS-R by treatment group	NA	Significant improvement in ADHD symptoms in atomoxetine group vs. placebo (d = 0.99; *p* <0.001); no significant difference in reduction of depressive symptoms (d = 0.20; ns)	1 −
		Guanfacine							
Biederman, 2008 [45]	Subgroup of 80 adolescents (gender for subset not reported) aged 13–17 years with ADHD (DSM-IV)		8 weeks, RCT, DB, PC; parallel groups: GXR (2, 3, and 4 mg/day, fixed dose escalation) or placebo	Not reported	Not reported for the adolescent subgroup	Mean change over time on parent-reported ADHD-RS-IV by treatment group	NA	No significant difference between GXR groups and placebo in reduction of ADHD symptoms (*p* > 0.05)	1 −
Sallee, 2009 [46]	Subgroup of 80 adolescents (gender for subset not reported) aged 13–17 years with ADHD (DSM-IV-TR)		9 weeks, RCT, DB, PC; parallel groups: GXR (1, 2, 3, and 4 mg/day, fixed dose escalation) or placebo	Not reported	Not reported for the adolescent subgroup	Mean change over time on parent-reported ADHD-RS-IV by treatment group	NA	No significant difference between GXR groups and placebo in reduction of ADHD symptoms (*p* = 0.20)	1 −
Wilens 2015 [47]	314 adolescents aged 13–18 years (mean 14.5 years; 64.7% male) with ADHD (DSM-IV-TR)		13 weeks, RCT, DB, PC; parallel groups: GXR (4 to 7 mg/day dependent on weight and optimal dose titration) or placebo	Subjects in behavioral treatment at study enrollment could continue their treatment	GXR: 74.5%; placebo: 70.1%	Mean change over time on adolescent-reported ADHD-RS-IV by treatment group	NA	Significant improvement in ADHD symptoms in GXR group vs. placebo (d = 0.52; *p* < 0.001)	1 −

Note: Pelham 2013 and Newcorn 2017a and 2017b are three-armed studies that investigated two active medications and placebo. ADHD—attention deficit/hyperactivity disorder; SUD—substance use disorder; CD—conduct disorder; RCT—randomized controlled trial; DB—double blind; SB—single blind; PC—placebo controlled; Tx—treatment; OROS-MPH—osmotic-release oral system methylphenidate; MPH-SODAS—extended release formulation of methylphenidate; MTS—methylphenidate transdermal system; IR-MPH—immediate release methylphenidate; LDX—lisdexamfetamine dimesylate; MAS-XR—mixed amfetamine salts extended release; GXR—guanfacine extended release; CBT—cognitive behavioral therapy; MET—motivational enhancement therapy; DSM-IV —Diagnostic and Statistical Manual of Mental Disorders; ADHD-RS-IV—DSM-IV ADHD Rating Scale; SNAP-IV—Swanson, Nolan, and Pelham Scale, version IV; CHI —Conners Hyperactivity-Impulsivity scale; IOWA-CRS—IOWA Conners Rating Scale; CDRS-R—Children’s Depression Rating Scale-Revised; CGI—Clinical Global Impression scale; TLFB—Time Line Follow-Back calendar method; NA—not applicable; ns—not significant. Mechanisms of action for each pharmacological treatment mentioned: Methylphenidate: increases dopaminergic and noradrenergic activity in the prefrontal cortex by inhibiting dopamine and noradrenaline reuptake. Pemoline: increases dopaminergic activity by blocking dopamine reuptake. Atomoxetine: not fully known but thought to be related to increased noradrenergic activity in the prefrontal cortex via selective noradrenaline reuptake inhibition. (Lis)dexamfetamine/mixed amfetamine salts: increases monoamine neurotransmitter release (including dopamine, norepinephrine, and serotonin) and prevents their reuptake. Guanfacine: not fully known but thought to be related to the stimulation of alpha2 adrenergic receptors in the brain.

**Table 2:** Risk of bias in the included studies on pharmacological interventions.

Study	Bias Arising from/Due to:					
	Rnd	Int	Mis	Mea	Sel	Overall
Adolescents with ADHD + SUD						
Riggs, 2011 [37]	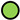	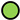	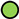	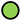	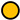	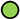
Szobot, 2008 [36]	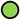	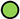	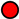	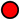	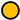	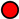
Riggs, 2004 [38]	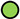	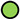	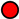	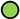	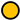	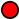
Thurstone, 2010 [39]	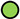	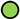	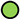	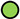	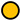	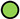
Adolescents with ADHD						
Pelham, 2013 [33]	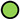	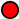	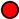	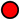	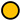	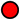
Findling, 2010 [27]	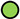	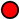	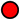	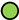	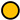	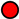
Wilens, 2006 [40]	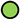	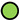	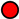	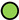	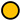	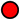
Findling, 2011 [42]	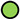	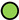	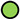	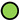	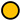	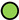
Spencer, 2006 [41]	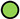	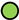	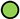	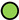	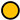	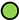
Bostic, 2000 [43]	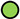	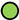	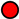	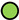	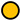	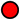
Bangs, 2007 [44]	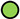	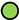	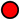	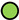	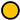	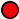
Biederman, 2008 [45]	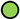	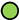	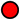	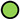	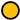	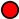
Sallee, 2009 [46]	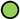	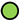	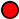	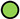	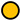	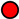
Wilens, 2015 [47]	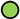	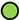	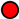	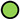	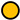	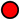
Newcorn, 2017a [34]	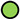	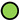	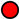	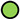	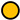	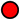
Newcorn, 2017b [34]	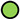	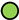	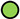	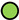	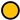	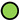

Note: 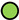 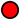 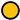 low risk/high risk/some concerns. Rnd—randomization proces; Int—intended interventions; Mis—missing outcome data; Mea—measurement of the outcome; Sel—selection of the reported result.

**Table 3:** Controlled studies (*n* > 40) of psychosocial treatment in adolescents with ADHD published since 2000.

AUTHOR, YEAR	Population	Study Design	Intervention	Treatment Completers	Outcome Measures	ADHD Outcomes	Functional Outcomes	Quality Rating
Clinic interventions: adolescent-focused								
Boyer, 2015 [68]	159 adolescents with ADHD (DSM-IV-TR) aged 12–17 years (mean 14.4; 74% male) 78% medicated (only MPH. stable). No significant differences in medication use at baseline, post-test, and 3-month follow-up, and no differences in number of adolescents receiving additional psychosocial treatment between end of treatment and follow-up.	2-arm RCT, 8-week treatment duration, stratified randomization on gender, medication (y/n), DB, 8 sessions of Plan My Life (PML) or 8 sessions Solution-Focused Treatment (SFT).	PML, *n* = 83, 8 adolescent sessions (frequency NR) CBT-based planning skills training, 2 parent sessions. SFT, *n* = 76, SFT = 8 adolescent sessions of self-formulated problem solving. Both PML and SFT also contained psychoeducation, MI, and personal treatment goal setting/monitoring and rewards.	ITT analyses End of treatment: PML: 79 (95%), SFT: 67 (88%), no significant differences in number of completers between conditions. Follow-up: PML: 77 (93%), SFT: 59 (71%), higher drop-out in SFT at follow-up.	ADHD-symptoms with Disruptive Behavior Disorder (DBD) rating scale (Pelham, et al., 1992) parent report. Planning and executive functions (BRIEF, Gioia, 2000). Neuropsychological measures (computer tasks).	No significant interactions between time and group were found on reported ADHD.	No differences between conditions at the end of treatment and 2-month follow-up.	1 −
Sprich, 2016 [73]	46 adolescents with ADHD (DSM-IV) aged 14–18 years (mean 15.1; 78% male), all on stable medication before randomization but changes allowed (*n* = 15), or all on stable medication but use varied. Weekly medication monitoring. 78% reported medication use at baseline and 84% at post-treatment. Group differences and control for medication or other therapy not reported.	2-arm cross-over RCT, SB, 4-month treatment duration, stratified randomization based on sex and ADHD severity, 12 sessions of CBT (approximately 17 weeks) vs. Wait-List (WL) control, assessments at the beginning and end of treatment and 4-month follow up (but unclear comparisons at follow-up).	CBT, *n* = 24, 7 modules over 12 individual sessions with a therapist (psychoeducation, organization/planning, distractibility, and adaptive thinking procrastination). Parents were included for 10 min at the end of each session (psychoeducation and goal-setting). In addition, two optional parent-only sessions (parenting style and contingency management). 94% of study completers participated in all 12 sessions. For the treatment completers, the average completion time was 17.3 weeks. WL, *n* = 22, no treatment for four months.	ITT analyses End of treatment (4 months after baseline): CBT: 21% (88%), WL: 22 (100%).	Independent evaluator administered parent and adolescent DSM-IV ADHD rating scales (Barkley 1990). Clinical Global Impression (CGI, NIMH, 1985). Categorical responder status (Steele, 2006).	Controlled effect sizes (d_corr_) comparing CBT vs. WL on parent-rated and adolescent-rated ADHD total-score symptom score at the end of treatment were 0.5 and 0.43, respectively. Article reports group differences tested in one analysis based on cross-over design, but comparisons are unclear.		1 −
Vidal, 2015 [74]	119 adolescents with ADHD (DSM-IV) aged 15–21 years (mean 17.2; 68% male), 100% medicated, stabilized doses for at least 2 months. *n* = 12 discontinued medication between pre-test and follow-up. No differences between conditions. Medication use and absence of other interventions was monitored weekly.	2-arm RCT, SB, treatment duration NR, group CBT (12 sessions) vs. Wait-List (WL) control group, assessments at baseline and at the end of treatment, assessments of control and intervention group coincided.	CBT, *n* = 59, participants received 12 manualized group sessions provided by a trained clinical psychologist (*n* = 2) based on CBT and MI, including psychoeducation (1 session), impulsivity/motivation (5 sessions), and planning strategy/attention (6 sessions). Parents were not involved. WL, *n* = 60, only monitoring of adherence and continuation of medication, no CBT or other psychological treatment. All participants were monitored weekly for medication adherence and the absence of other treatments.	ITT analyses End of treatment: CBT: 45 (76%), WL: 44 (73%).	ADHD by Adolescent and Parent reports (ADHD-RS, DSM-IV: Dupaul, 1998), CGI (NIMH, 1985), Weiss Functional Impairment Rating Scale, CADDRA 2000). Depression (BDI, Beck, 1941), Anxiety (STAI, Spielberger, 1986).	Controlled effect sizes (d_corr_) comparing CBT vs. WL on parent-rated and adolescent-rated ADHD total-symptom scores at the end of treatment were 1.06 and 0.99, respectively.	Participants in the CBT group showed significant improvement in parent-reported functional impairment compared with participants in the WL condition. No significant differences on self-reported functional impairment, anxiety, and depression.	1 −
Meyer, 2021 [69]	184 adolescents with ADHD (DSM-5) aged 15–18 years received Structured Skills Training Group (SSTG): mean age 16.5 years (SD = 0.88), 34% males; control group: mean age 16.7 (SD = 0.94), 38% male. 77% received ADHD medication; 36% received additional medication. Current ADHD medication assessed at baseline, post-treatment, and follow-up by parent report. Pharmacological treatment needed to be stable for study inclusion and participants were requested not to take part in any other psychological treatment during the study. 18–21% underwent major changes in medication during study. No significant differences.	2-arm RCT, SB, treatment duration NR, SSTG group-based dialectical behavior therapy (DBT) originally developed for adults with ADHD vs. psychoeducation control group (Control). Baseline assessment 2 weeks before treatment, with post-treatment assessment at 2 weeks and 6 months after treatment.	SSTG, *n* = 93, participants received 14 weekly manualized group sessions of 2h each provided by a trained clinical psychologist (*n* = 2), including psychoeducation, strategies managing ADHD, DBT elements, and homework assignments. CONTROL, *n* = 91, manual-based psychoeducation group program (SKILLS), 3 2-h sessions including information about relevant ADHD-related issues (symptomatology, strengths and challenges, sleep and diet, stress management, etc.) and a book with tools facilitating schoolwork. Average treatment fidelity was only measured for SSTG, and was considered acceptable to good.	ITT analyses SSTG: Baseline: 85 (91%), Post-treatment: 74 (80%), Follow-up: 71 (76%), Included in analyses: 85 (91%). CONTROL: Baseline: 79 (87%), Post-treatment: 61 (67%), Follow-up: 57 (63%), Included in analyses: 79 (87%).	Primary outcomes: Adult ADHD self-report scale for adolescents (ASRS-A) Sonnby et al., 2015). Self- and parent-rated functional impairment by Child Sheehan Disability Scale (CSDS; Whiteside, 2009). Impact of ADHD symptoms (IAS) on well-being; scale constructed for this study. Global Quality of Life scale (GQL, Ivarsson, 2010). Five Facet Mindfulness Questionnaire (FFMQ, Ba2r, 2008). Secondary measures included questionnaires on behavioral and emotional problems, stress, anxiety, and sleep problems.	No between group-differences in improved ADHD-symptoms. Only significant within-group effects: Moderate effects for parent-reported ADHD symptoms in the SSTG group (d = 0.59 (T1–T2), d = 0.62 (T1–T3)).	No between group-differences; small within-group differences (d = 0.26–0.45).	1 −
Clinic interventions: parent/Family-focused								
Barkley, 2001 [67]	97 adolescents with ADHD (DSM-IV) aged 12–18 years (mean: not reported; 90% male) Criteria: stable medication, no other social therapies. Medication, reported for all assessments, 56% medicated at baseline. No significant differences in medication use at baseline, end of treatment, and follow-up.	2-arm RCT, 9 week treatment duration, sequential randomization to Problem solving Communication Training (PCT) or PCT + Behavior Parent Training (PCT + BPT), assessments at baseline and at the end of treatment.	PCT, *n* = 58, 18 twice-weekly sessions of problem solving, communication training, and cognitive restructuring). PCT + PBT, *n* = 39, 9 twice-weekly sessions PCT followed by 9 twice-weekly sessions PBT (positive parenting, point system/contingency management).	No ITT analyses End of treatment: PCT: 36 (62%), PCT + PBT: 32 (82%). Follow-up: PCT: 33 (57%), PCT + PBT: 29 (74%). Higher drop-out in PSCT than in BMT/PSCT.	ADHD/ODD DSM-IV Raing Scale (DuPaul et al., 1998), parent, adolescent and teacher report Conflict Behavior Scale (CBQ, Prinz, Foster, Kent, and O’Leary, 1979) and other conflict measures.	No significant interactions between time and group were found on reported ADHD.	No differences between conditions at the end of treatment and at 2-month follow-up.	1 −
Sibley 2016 [70]	128 adolescents with ADHD (DSM-IV-TR) aged 11–15 years (mean = 12.8; 65% male, mostly Hispanic), additional medication and other treatment use was allowed. 34% used medication (no significant differences between conditions at post-test and follow-up, no differences in participants who changed dose or started new medication), 6.4% received individual therapy, no group differences in use of other interventions (academic tutoring, educational accommodation, and individual therapy)	2-arm RCT 10 week treatment duration, stratified randomization on medication status and oversampling of Supporting Teen’s Academic Needs Daily (STAND) compared with treatment as usual (TAU), assessments pre/post intervention (timing unclear) and at 6 months follow-up.	STAND, *n* = 67, received 10 family therapy sessions, 50 min with parents and teens by MI- and STAND trained clinicians. Treatment includes 4 selections out of 7 modular MI and CBT-based sessions to train academic/organization/problem solving skills. In addition, parents were invited to 4 group sessions, but these were not well attended (22–52% per session). TAU: *n* = 61, families were encouraged to seek services in the community.	ITT analyses End of treatment: STAND: 60 (90%), TAU: 55 (90%). Follow-up: STAND: 55 (82%), TAU: 51 (84%). Missing data per condition NR. 95% post-test data available from at least two sources, at follow-up this was 87%. No differences between completers (at least one source) and non-completers. Pre-treatment differences on IQ and ADHD-subtypes (included as covariates).	ADHD (DBD, Pelham, 1992). OTP and Grade Point Average (GPA) defined as primary outcome measures. Parent and teacher ratings of OTP (AAPC, blinded ref). Official school grades (electronic gradebook, Quarterly GPA) Parent-rated Parent-Teen conflict (CBQ-20, Prinz 1979) Parenting stress (CSQ, Brannan, 1997) Parent OTP involvement (PAMS, blinded ref) Recorded homework Bookbag organization (Evans, 2009).	Controlled effect sizes (d_corr_) comparing STAND vs. TAU on parent-rated ADHD total-score symptom score were 0.72 at the end of treatment and 0.59 at follow-up. No differences on teacher-rated ADHD.	Stronger improvements at post-test and FU on parent-rated OTP, parenting problems and homework for STAND than TAU. No differences on GPA, teacher-rated outcomes, and adolescent-rated parent−teen conflict.	1 −
Sibley 2019 [71]	123 adolescents with ADHD (DSM-5) aged 11–17 years (mean 13.6; 80% male), clinic-based treatment but recruitment via schools, and additional medication and other treatment use was allowed. 42% used medication at baseline (no significant differences across assessments), and medication use was controlled and monitored during data-collection and controlled for in analyses.	2-arm RCT, treatment duration 10–12 weeks, randomization in 20 waves to Dyadic Supporting Teen’s Academic Needs Daily (STAND) or Group STAND, assessments at baseline and at the end of treatment and at 6-month follow-up.	Dyadic STAND, *n* = 63 received 10 weekly parent−teen dyadic therapy sessions, 60 min by MI- and STAND trained clinicians (see Sibley 2016). Group STAND: *n* = 60 received 8 weekly group sessions, consisting of 75 min of separate group sessions for parents and adolescents, and 15 min of final blended parent−teen group session (total 90 min) by trained clinicians. Therapy dose and fidelity scores did not differ between interventions. However, MI integrity was higher in Dyadic than Group STAND, but Group STAND showed higher levels of MITI giving information than Dyadic STAND, and parents in Group STAND reported a greater self-efficacy and normalization of their difficulties than parents in the Group STAND.	ITT analyses End of treatment: Dyadic STAND: 59 (94%), TAU: 58 (97%). Follow-up: STAND: 50 (79%), TAU: 55 (92%). Differences not tested.	ADHD (DSM-5 ADHD-rating scale, Sibley and Kuriyan, 2016). OTP and Grade Point Average (GPA) defined as primary outcome measures. Parent and teacher ratings of OTP (AAPC, blinded ref). Official school grades (electronic gradebook, quarterly GPA). Parent-rated Parent−Teen conflict (CBQ-20, Prinz 1979). Parenting stress (CSQ, Brannan, 1997). Parent OTP involvement (PAMS, blinded ref). Recorded homework Bookbag organization (Evans, 2009).	No significant differences in treatment outcomes between conditions. Parents with elevated ADHD-scores and at least moderate depression symptoms and high conflict dyads benefitted more from the Dyadic than group-based STAND.	No between-group differences on the other functional outcomes reported.	1 −
Sibley 2020 [72]	287 adolescents with ADHD (DSM-5) aged 11–17 years (mean 14.0; 71% male), clinic-based treatment at community clinics, with additional medication allowed but monitored and controlled for in the analyses. Of the participants in the intervention group, 31.2% used medication at baseline compared with 23.6% in the control group (usual care). From baseline to post-treatment: increased medication use in the intervention group compared with usual care.	2-arm RCT, treatment duration varied (only mean session reported per condition), stratified randomization procedure within agency to either Teen’s Academic Needs Daily (STAND) or Usual Care (UC) Baseline assessment, post-test at 16 weeks post baseline and follow-up at 12 weeks after the post-treatment assessment.	STAND: *n* = 138, 10 weekly parent−teen dyadic therapy sessions, 60 min by STAND trained clinicians (see Sibley 2016). Received number of sessions: 13.99 (SD = 13.80). UC: *n* = 140, received mean 17.38 (SD = 15.26) weekly therapy sessions of usual care. Coding of 78 available UC audio tapes using STAND fidelity checklists indicated high treatment differentiation (53.8% of items were not present on any UC recordings).	ITT analyses End of treatment: STAND: 114 (83%), UC: 111 (79%). Follow-up: STAND: 112 (81%), TAU: 106 (76%). Differences were not significant.	Primary outcomes: Parent and teacher reports ADHD-symptoms (Conners-3, 2008); parent and teacher DSM-5 ADHD checklists (Sibley and Kuriyan, 2016). Secondary outcomes: Academic impairment: OTP and Grade Point Average (GPA) Parent and teacher ratings of Adolescent Academic Problems Checklist (AAPC, blinded ref). Family impairment: Parent and adolescent rated Conflict Behavior Questionnaire-20 (CBQ-20). Disciplinary incidents: Counts of all disciplinary incidents (e.g., detention, in-school suspension) during academic quarter immediately preceding each assessment.	No significant differences in improved treatment outcomes between conditions.	No between-group differences on other functional outcomes reported.	1 −
School interventions: adolescent-focused								
Evans 2007 [62]	79 adolescents with ADHD (DSM-IV-TR) aged 10–14 years (mean 11.9; 77% male). Prevalence of medication use examined in analyses, but prevalence and group differences NR.	2-arm RCT, treatment duration NR, cluster-randomization per school (*n* = 5, 2:2, 1 extra school added to the control group), 15 sessions of training and consultation model of the Challenging Horizons Program (CHP-C) or “treatment advice” (Control), assessments pre- and post-treatment (after 6 months) and at 12-, 18-, 24-, and 30-months of follow-up.	CHP-C, *n* = 42, 15 sessions targeting academic and social skills, individual guidance by mentors. Monthly medication monitoring (if symptoms above threshold, option for additional medication or psychosocial treatment, 92% opted for additional psychosocial interventions). Control, *n* = 37, parents received summaries of baseline intake, a list of local treatment providers, and could pursue treatment of their choice.	ITT-analyses NR, but Hierarchical Linear Modeling have been ITT. Reported Year 3 completion rates do not match N final evaluation. Year 1: CHP-C: 40 (95%), Control: 32 (88%). Year 2: CHP-C: 37 (88%), Control: 25 (66%). Year 3: CHP-C: 32 (76%), Control: 22 (61%).	ADHD, parent-report (DBD, Pelham, 1992). Behavior Assessment System for Children (BASC; Reynolds and Kamphaus, 1993). Impairment Rating Scale (IRS, Fabiano, 2006). Grades Social Skills Rating System (SSRS, Gresham and Elliot, 1990).	Results of the analyses suggest small cumulative benefits of CHP-C on inattention.	Small cumulative benefits of CHP-C on social functioning.	1 −
Evans 2016 [63]	326 adolescents with ADHD (DSM-IV-TR), grades 6–8 (mean: NR; 70% male). 43–52% medicated at baseline, no group differences.	3-arm RCT at nine schools, treatment duration 1 year, stratified randomization for site and medication at baseline. 1 (academic) year of the afterschool Challenging Horizons Program (CHP-AS) or CHP-M or “treatment advice” (control).	CHP-AS, *n* = 112, twice per week group-based afterschool program targeting social impairment, education/study skills group. Individual guidance/monitoring by a primary counselor (PC). CHP-M, *n* = 110, weekly individual mentor meetings monitoring and delivery of subset of the CHP-AS interventions. CC, *n* = 104, summary report of intake evaluation on request and list of local treatment providers.	ITT analyses End of treatment: CHP-AS: 104 (91%), CHP-M: 108 (98%), CC: 104 (100%), Follow-up: CHP-AS: 104 (95%) CHP-M: 108 (%) CC: Higher treatment drop-out in CHP-AS (22% of 112 or 105, not clear) compared with CHP-M (3% of 110/108 not clear). Treatment-drop-out in CC not reported.	ADHD, parent-report (DBD, Pelham, 1992). Organization, time-management, planning skills (COSS: Abikoff, 2000). Homework problems (CPS, Evans, 2012). Overall academic functioning (HPC, Anesko, 1987). Interpersonal functioning (IRS, Fabiano, 2006).	Only the interaction between time and group for parent-rated inattention symptoms was significant. Compared with Control, CHP-AS only showed stronger benefits for parent-rated inattention symptoms (no differences on parent-rated hyperactivity and teacher ADHD-ratings). Compared with CHP-M, CHP-AS showed stronger improvements in parent-rated inattention at follow-up assessment only.	Compared with Control, CHP-AS showed stronger benefits on organization/planning skills, homework, and academic skills. No differences for social and teacher-rated academic functioning. Compared with CHP-M, CHP-AS showed stronger improvements on task planning and academic functioning at the follow-up assessment only.	1 −
Langberg 2012 [64]	47 adolescents with ADHD (DSM-IV) aged 11–14 years (mean: not reported; 55% male). 66% medicated.	2-arm RCT, treatment duration 11 weeks, Homework Organization and Planning Skills (HOPS) intervention with Waiting list-control group (WL), assessments at baseline and end of treatment 3-month follow-up in intervention group.	HOPS, *n* = 23, 16 individual school-day bi-weekly/weekly 20 min sessions over 11-week period on school materials organization, homework recording and management, and planning/time management including skill tracking with a point/reward system. Two 1 h parent meetings with students. WL: waiting list control group	ITT, but no info about attrition or treatment drop-out. Seems all participants completed the interventions and assessments, but could also be as selection? HOPS was provided between 11–19 weeks (M = 13.8, Med = 14).	No primary/secondary outcomes defined. Homework (parent-rated HPC, Ramirez, 1987). Organizational skills (COSS, parent, teacher, and student report, Abikoff, 2008). Parent-rated ADHD (DSM-IV-based scale VADPRS, Vanderbildt) Parent Skills Implementation Questionnaire.	No differences between HOPS intervention and WL control on ADHD (interaction, *p* = *0*.059).	HOPS intervention resulted in a stronger improvement of organizational skills than for the WL control.	1 −
Schramm 2016 [65]	*n* = 113 adolescents with ADHD (DSM-IV-TR) aged 12–17 years (mean = 14.0; 85% male). No selection criteria for medication use and other interventions. 50.4% medicated, lower medication use in 38.9% in waiting list control group compared with the training intervention group (60%) and active control group (51.4%).	3-arm RCT, treatment duration approximately 3–6 months, stratified for gender, 6-month CBT-based adolescent-directed problem solving and organizational skills training (INT) or 6-month progressive muscle relaxation (active control (AC)) or 3-month waiting-list control group (WL), assessments at baseline and at the end of treatment.	INT: *n* = 40, participants received CBT-based individual training sessions provided by trained special education or psychology students (max. 20, 60 min) combined with a behavioral component based on contingency management by parents and teachers. AC, *n* = 36, participants received twice-weekly group (4–5 pp) sessions of PMR (12–15 sessions, 60 min). WL, *n* = 37	ITT-based last observation carried forward. End of treatment: INT: 39 (98%), AC: 34 (94%), WL: 36 (97%). Overall, 6.4% missing data (higher in teacher reports).	ADHD DSM-IV symptom Checklists (SBB-HKS, Dopfner, 2007). Parent and teacher reports. Hyperactivity-subscale SDQ (Goodman, 1997). Academic Enablers (AVL, Lauth, 2004). Meta-cognitive skills (WSW, reading strategies, Schlagmuller, 2007). Int/Ext problems, alertness, flexibility, inhibition, self-rated outcomes on ADHD, etc.	Compared with WL, INT resulted in stronger reductions of parent and teacher-rated ADHD, teacher-rated learning behavior, internalizing problems, and self-rated learning problems compared with WL. No differences between INT and PMR.	Compared with WL, INT resulted in stronger reductions of teacher-rated learning behavior, internalizing problems, and self-rated learning problems. No differences between INT and PMR.	1 −
School interventions: Parent/Family-focused								
Steeger 2016 [66]	*n* = 104 (randomized from *n* = 108) adolescents with ADHD (DSM-IV) aged 11–15 years (mean 12.5; 69% male). 84% medicated Criteria: stable medication, 84% medicated at baseline, and no significant differences between groups.	4-arm RCT: 2 × 2 mixed group factorial design, treatment duration of 5 weeks, randomization to Cogmed Working Memory Training (CWMT) and group Behavioral Parent Training (BPT) or to CWMT and Control-BPT (CNT-BPT) or to Control-CWMT (CNT-CMWT) and BPT, or to CNT-CMWT and CNT-BPT, assessments at baseline and at the end of treatment.	CWMT + BPT, *n* = 26, CWMT: 25-day high-dose adaptive computerized WM training (Cogmed). BPT: 5-week group BPT program based on COPE aimed at mother−adolescent interactions, adolescent compliance, and maternal control, reducing conflict and adolescent ODD. CNT-CWMT + BPT, *n* = 26, 25 day low-dose non-adaptive computerized WM training (Cogmed) + BPT: see above. CWMT-CNT-BPT: *n* = 26, 5 CWMT: see above. CNT-BPT: active control program of didactic lectures on adolescent development, homework of weekly readings self-help guide. No facilitation of practice/ feedback. CNT-CWMT + CNT-BPT, *n* = 26, CNT-CWT: see above. CNT = BPT: see above.	No ITT analyses but analyses on completers-only (*n* = 96), excluding participants with IQ < 70 (*n* = 3) and participants with mothers with <75% BPT attendance (*n* = 2), final sample *n* = 91. End of treatment: CWMT + BPT, *n* = 22 (85%), CWMT + CNT-BPT, *n* = 23 (88%), CNT-CWMT + BPT, *n* = 25 (96%), CWMT + CNT-BPT, *n* = 26 (100%).	Mother and teacher ratings of ADHD Rating Scale-IV (ADHD-RS, DuPaul 1998). Executive functioning (BRIEF, Gioia, 2000). Mother-reported: Parenting behavior (APQ, Frick 1991). Mother–adolescent conflict (CBQ, Robin, 2002). Oppositional behaviors (CBCL, Achenbach, 2001).	No significant differences between conditions on ADHD-symptoms and parenting variables.	No significant differences between conditions on parenting variables. Interaction effect on global functioning showed better outcomes of participants in the control-CWMT + BPT group.	1 −

**Table 4:** Risk of bias in the included studies on psychosocial and complementary intervention.

	Bias Arising from/Due to:	Rnd	Int	Mis	Mea	Sel	Overall
	Psychosocial Interventions						
1	Barkley, 2001 [67]	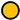	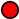	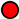	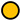	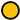	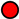
2	Boyer, 2015 [68]	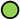	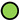	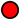	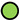	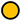	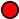
3	Evans, 2007 [62]	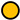	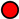	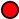	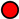	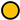	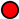
4	Evans, 2016 [63]	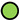	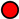	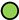	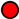	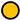	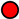
5	Langberg, 2012 [64]	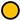	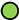	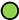	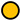	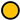	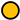
6	Meyer, 2021 [69]	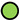	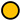	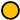	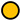	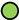	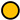
7	Schramm, 2016 [65]	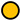	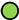	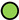	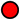	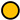	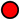
8	Sibley, 2016 [70]	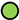	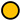	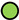	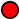	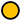	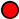
9	Sibley, 2019 [71]	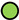	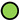	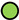	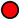	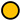	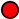
10	Sibley, 2020 [72]	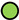	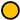	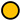	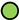	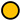	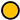
11	Sprich, 2016 [73]	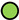	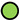	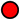	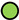	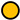	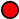
12	Steeger, 2016 [66] *	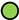	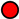	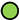	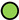	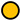	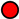
13	Vidal, 2015 [74]	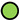	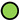	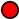	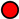	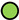	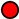
	**Complementary Interventions**						
14	Ahmed, 2011 [75]	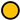	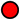	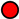	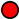	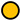	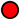
15	Bink, 2016 [76]	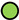	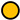	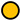	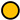	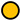	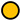
16	Gray, 2012 [77]	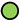	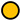	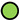	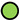	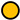	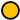
17	Matsudaira, 2015 [78]	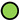	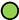	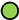	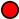	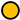	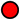

Note: 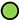 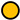 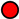: green = low risk of bias, orange = some concerns and red = high risk of bias. Rnd—randomization process; Int—intended interventions; Mis—missing outcome data; Mea—measurement of the outcome; Sel—selection of the reported result. * Since Steeger, 2016, examined both a psychosocial and a complementary intervention, this trial was included in the review of both psychosocial and complementary interventions.

**Table 5:** Controlled studies (*n* > 20 per condition) of complementary interventions in adolescents with ADHD published since 2000.

Author, Year	Population	Study Design	Intervention	Treatment Completers	Outcome Measures	ADHD Outcomes	Functional Outcomes	Quality Rating
Gray, 2012 [77]	60 adolescents aged 12–17 years (mean 14.3; 87% male) with ADHD (DSM-version not reported) and learning disabilities, 98% medicated.	8-week SB, RCT unbalanced randomization (3:2 assignment to the working memory (WM) training), 5-week working memory training program (WM), or 5-week mathematics training program (MT) (unbalanced randomization). Post-test assessment 3 weeks after the end of treatment.	WM, *n* = 36, 45-min training sessions of Cogmed at school, 4–5 days a week for 5 weeks. MT, *n* = 24, 45-min training sessions of mathematics training program (Academy of Math; (Torlakovic, 2011), 4–5 days a week for 5 weeks.	ITT analyses WM: 32 (89%), MT: 20 (83%).	ADHD-symptoms were assessed with the Strengths and Weakness of ADHD-symptoms and Normal-behavior scale (SWAN, Swanson et al., 2001) and the IOWA Conners scale (Pelham et al., 1989). Academic Progress (WRAT-4PM; Roid and Ledbetter, 2006). WM and attention measures, i.e., CANTAB.	No group differences on ADHD and other measures. Only differences on WM criterion measures.		1 −
Steeger, 2016 [66]	*n* = 104 (randomized from *n* = 108), adolescents with ADHD (DSM-IV) aged 11–15 years (mean 12.5; 69% male), 84% medicated.	7-week RCT with four conditions: 5-week Cogmed working memory training (CWMT) combined with behavioral parent training (BPT), 5-week CWMT with a control parent intervention (CNT-BPT), 5-week control version of CWMT (CNT-CWMT) combined with BPT, or 5-week CNT-CWMT combined with CNT-CBT.	CWMT + BPT, *n* = 26, CWMT: 25-day high-dose adaptive computerized WM training (Cogmed). BPT: 5-week treatment group BPT program based on COPE aimed at positive mother−adolescent interactions, adolescent compliance, and maternal control, reducing conflict and adolescent ODD. CNT-CWMT + BPT, *n* = 26, 25-day low-dose non-adaptive computerized WM training (Cogmed) + BPT: see above. CWMT-CNT-BPT: 5 CWMT: see above. CNT-BPT: Control parent intervention of didactic lectures on adolescent development and homework of weekly readings from self-help guide. No facilitation of practice or feedback. CNT-CWMT + CNT-BPT, *n* = 26, CNT-CWT: see above. CNT = BPT: see above.	No ITT-analyses, but analyses on completers-only excluding participants with IQ < 70 and participants with mothers with <75% BPT attendance, final sample, *n* = 91. *n* = 108 included, *n* = 104 randomized, and *n* = 8 dropped out. Drop-out = 8% of 104 pp, higher drop-out in CWMT than in CWMT-CNTR.	ADHD Rating Scale-IV (ADHD-RS, DuPaul, 1998) mother and teacher report. Executive functioning (BRIEF, Gioia, 2000). Mother-reported: Parenting behavior (APQ, Frick, 1991). Mother–adolescent conflict (CBQ, Robin, 2002). Oppositional behaviors (CBCL, Achenbach, 2001).	No significant differences between conditions on ADHD-symptoms and parenting variables.	No significant differences between conditions on parenting variables. Interaction effect on global functioning showing better outcomes of participant in the control-CWMT + BPT group.	1 −
Bink, 2016 [76]	90 adolescents with ADHD (DSM-IV-TR) aged 12–24 years (mean 16.0; 100% male), 49–52% medicated, no between group differences in medication use at baseline and follow-up.	1-year un-blinded, RCT unbalanced randomization (2:1) stratified randomization for age groups of 12–15, 16–20, and 21–24 years. 25-week neurofeedback training (NF) + treatment as usual (TAU) or 25-week TAU.	NF + TAU, *n*= 59, 25 weeks of 2–3 weekly 30 min training sessions of a theta/SMR training (Lubar 2003) + At least 5 weeks TAU consisting of regular cognitive–behavioral therapy, systemic therapy, and/or supportive counselling for the adolescent and/or his parent(s). TAU: *n* = 31, at least 25 weeks of TAU (see above).	ITT analyses on *n* = 87 (*n* = 56 NF + TAU, *n* = 31 TAU) End of treatment NF + TAU: 45 (76%) TAU: 26 (85%) 1-year follow-up 41 (73%) TAU: 19 (61%)	MINI ADHD-subscale (Sheehan et al., 1998). ADHD Rating Scale-IV (ADHD-RS, DuPaul, 1998). Youth Self-Report (YSR, Achenbach, 1991). Neuropsychological measures (computer tasks).	No significant differences between NF + TAU and TAU on all outcome measures.		1 −
Matsudaira, 2015 [78]	75 adolescents with ADHD (DSM-IV) aged 12–16 years (mean 13.7; 100% male), 19.7% psychostimulant medication. No differences between conditions.	12 weeks, DB, RCT placebo-controlled, stratified randomization by day/boarding school and age group (12–14 years and 15–17 years). Long chain-polyunsaturated fatty acid (LC-PUFA) with placebo.	LC-PUFA, *n* = 38, 12 weeks of daily dose of six LC-PUFA capsules of omega-3 fatty acids (EPA 558 mg and DHA 174 mg), omega-6 fatty acid У-linoleic acid 60 mg, and vitamin E 9.6 mg (in the natural form, α-tocopherol). Placebo, *n* = 38, 12 weeks of daily dose of placebo (medium chain triglycerides).	“ITT” analyses on *n* = 69 (LC-PUFA, *n* = 33, Placebo, *n* = 36) Per protocol on *n* = 50 End of treatment: LC-PUFA: 23 (61%) Placebo: 27 (71%)	ADHD measured by Conners’ Teacher Rating Scales (CTRS-L), which assessed each of 59 items of child behavior on a four-point scale (Conners et al., 1998).	No differences in ADHD ratings between LC-PUFA and placebo at 12-weeks of follow-up.		1 −
Ahmed, 2011 [75]	84 adolescents with ADHD (DSM-IV-TR) aged 11–16 years (mean = 13.8; 64% male), medicated: % not reported.	10-week RCT, no details on randomization. 10-week aerobic moderate intensity exercise program (MA exercise) or no intervention. Blinding not reported.	MA Exercise, *n* = 42, 10 weeks, 3 days a week 40–50 min aerobic sessions and home program parental instruction of 30 min outdoor walking in weekends. No exercise, *n* = 42, 10 weeks.	No information on completers/drop-out rates. No between group differences on physical characteristics (weight) and outcome measures.	Attention problems, motor skills, task orientation, emotional and oppositional behavior, and academic and classroom behavior: modified Conner’s Rating Scale (Conners, et al., 1998).	Stronger improvement in attention problems in participants who received the MA exercise program compared with the control group.		1 −

## Supplementary Materials

The following are available online at https://www.mdpi.com/article/10.3390/jcm10173908/s1. Figure S1: PRISMA_2020_checklist. Table S1: SIGN grading system. Table S2: Search terms and limiters. Table S3: Placebo-controlled studies of pharmacological treatment in adults with ADHD and comorbid SUD.
