# Supplementary material for: Treatment of Adolescents with Concurrent Substance Use Disorder and Attention-Deficit/Hyperactivity Disorder: A Systematic Review
Source: J Clin Med. 2021 Aug 30;10(17):3908. doi: 10.3390/jcm10173908 (PMC8432200; doi:10.3390/jcm10173908)
Supplement: Supplementary file 1 [file jcm-10-03908-s001.zip › jcm-1293750-supplementary.pdf]

Supp. Table S1. SIGN grading system (Harbour et al., 2001):

1 ++	High quality meta-analyses, systematic reviews of RCTs, or RCTs with a very low risk of bias
1 +	Well-conducted meta-analyses, systematic reviews, or RCTs with a low risk of bias
1 −	Meta-analyses, systematic reviews, or RCTs with a high risk of bias
2 ++	High quality systematic reviews of case control or cohort or studies, or<br>High quality case control or cohort studies with a very low risk of confounding or bias and a high probability that the relationship is causal
2 +	Well-conducted case control or cohort studies with a low risk of confounding or bias and a moderate probability that the relationship is causal
2 −	Case control or cohort studies with a high risk of confounding or bias and a significant risk that the relationship is not causal
3	Non-analytic studies, e.g. case reports, case series
4	Expert opinion

Table S2. Search terms and limiters

<u>Search terms in Medline</u>

ADHD:

1 “attention deficit and disruptive behavior disorders”
2 attention deficit disorder with hyperactivity.mp.
3 ADHD.tw,kw.
4 ADDH.tw,kw.
5 "attention deficit" ADJ2 "hyperactivity"
6 ADHS.tw,kw.
7 TDAH.tw,kw.
8 ((attention$ or behav$) adj3 (defic$ or dysfunc$ or disorder$)).tw,kw.
9 (impulsiv$ or inattentiv$ or inattention$).tw,kw.
10 (minimal adj3 brain adj3 (disorder$ or dysfunct$ or damage$)).tw,kw.
11 (hyperactiv$ or hyper-activ$).tw,kw.
12 ADHD
13 ADHD.mp. or exp Attention-Deficit-Hyperactivity Disorder/
14 hkd.mp.
15 exp Hyperkinesis/
16 hyperkine$.mp.
17 overactive.mp.
18 or/1-17

Psychostimulants:

1 exp Amphetamines/
2 (amphetamin$ or amfetamin$ or anfetamin$).mp.
3 benzedrin$.mp.

4 (dexamphetamin$ or dexamfetamin$ or dextroamphetamin$ or dextroamfetamin$ or dexedrin$).mp.
5 (dex-amphetamine or dex-amfetamine or dextro-amphetamine or dextro-amfetamine).mp.
6 (lis-dexamphetamin$ or lis-dexamfetamin$).mp.
7 mixed amphetamine salts
8 Lisdexamfetamine.tw,kw.
9 exp Methylphenidate/
10 methylphenidate.mp.
11 OROS$.tw,kw.
12 OROS.mp.
13 exp Dexmethylphenidate Hydrochloride/
14 Dexmethylphenidate.mp.
15 (Adderall or Amphetamine or Desoxyn* or Phenopromin or Amfetamine or Phenamine).ti,ab.
16 (Centramina or Fenamine or Levoamphetamine or Dexamfetamine or Dexamphetamine or Dexedrine).ti,ab.
17 (Dextroamphetamine or DextroStat or Oxydess or Methylamphetamine or Methylenedioxyamphetamine).ti,ab.
18 (Methamphetamine or Chloroamphetamine or Metamfetamine or Deoxyephedrine or Desoxyephedrine or Ecstasy).ti,ab.
19 (Concerta or Daytrana or Methylphenidate or Equasym).ti,ab.
20 (Methylin or Tsentedrin or Centedrin or Phenidylate or Ritalin$).ti,ab.
21 (Elvanse or Focalin or Dexmethylphenidate or Lisdexamfetamine or Vyvanse or Medikinet).ti,ab.
22 (Metadate or Quillivant).ti,ab.
23 or/1-23

Other medications:
1 exp Clonidine/
2 exp Bupropion/
3 exp Atomoxetine Hydrochloride/
4 exp Guanfacine/
5 exp Modafinil/
6 exp Pemoline/
7 exp Imipramine/
8 exp Desipramine/
9 (Atomoxetine or Biphentin or Bupropion or Amfebutamone or Zyntabac or Quomen or Wellbutrin or Zyban).ti,ab.
10 (Catapres* or Clonidine or Klofenil or Clofenil or Chlophazolin or Gemiton or Hemiton or Isoglaucon or Klofelin).ti,ab.
11 (Clopheline or Clofelin or Dixarit or Duraclon or Guanfacine or Estulic or Kapvay).ti,ab.
12 (Modafinil or Nexiclon or Strattera).ti,ab.
13 or/1-13

Psychosocial interventions:
1 "Contingency Management".af.
2 "Management techniques".af.
3 "contingency techniques".af.
4 "psychosocial interventions".af.
5 "psychosocial treatment".af.
6 "psychosocial therapy".af.
7 "social skills training".af.
8 "social skills intervention".af.
9 "social skills treatment".af.
10 "problem solving intervention".af.
11 "problem solving treatment".af.
12 "problem solving training".af.
13 "problem solving therapy".af.
14 "behavior modification".af.
15 "cognitive behavior treatment".af.
16 "cognitive behavior therapy".af.
17 "cognitive behavior training".af.
18 "parent training".af.
19 "parent counseling".af.
20 "parent support".af.
21 "school-based".af.
22 "classroom-based".af.
23 "school intervention".af.
24 "classroom intervention".af.
25 "teacher training".af.
26 "after-school".af.
27 "remedial teaching".af.
28 "peer tutoring".af.
29 "computer assisted learning".af.
30 "task modification".af.
31 "curriculum modification".af.
32 "classroom management".af.
33 "education intervention".af.
34 "multimodal$".af.
35 "multimodal treatment".af.
36 "multimodal therapy".af.
37 "multimodal intervention".af.
38 "educational intervention".af.
39 "verbal self-instruction training".af.
40 "cognitive training".af.
41 "cognitive remediation".af.
42 "social skills training".af.

43 "social skills education".af.
44 behavior therapy.af.
45 or/1-44

Complementary interventions:

1 "few foods diet".af.
2 "elimination diet".af.
3 "oligoantigenic diet".af.
4 "restriction diet".af.
5 "food intolerance".af.
6 "food allergy".af.
7 "food hypersensitivity".af.
8 "food color".af.
9 "food dye".af.
10 "Feingold diet".af.
11 dietary supplements.mp. or exp Dietary Supplements/
12 exp Fatty Acids/ or omega-3.mp.
13 "Kaiser Permanente diet".af.
14 "K-P diet".af.
15 "tartrazine".af.
16 "azo dye".af.
17 "carmoisine".af.
18 "sunset yellow".af.
19 vitamin.mp. or exp Vitamins/
20 "St John's Wort".af.
21 "yoga".mp. or exp Yoga/
22 exp Mindfulness-Based Interventions/ or exp Mindfulness/ or mindfulness.mp.
23 alternative medicine.mp. or exp Alternative Medicine/
24 "valerian".af.
25 "tryptophan".af.
26 "melatonin".af.
27 exp Acupuncture/
28 "acupuncture".af.
29 " Tai Chi".af.
30 exp Osteopathic Medicine/ or osteopathic medicine.mp.
31 chiropractic.mp.
32 exp Osteopathic Medicine/ or osteopathic medicine.mp.
33 chiropractic.mp.
34 "cognitive training".af.
35 "attention training".af.
36 "working memory training".af.
37 "cognitive remediation".af.

38 "executive function training".af.
39 "neurofeedback".af.
40 "EEG biofeedback".af.
41 "neurotherapy".af.
42 "slow cortical potentials".af.
43 neurofeedback.mp. or exp Biofeedback, Psychology/ or exp Neurofeedback/ or exp Electric Stimulation Therapy/
44 or/1-43

Controlled trials:
1 randomized controlled trial.pt.
2 controlled clinical trial.pt.
3 randomized.ab.
4 placebo.ab.
5 drug therapy.fs.
6 randomly.ab.
7 trial.ab.
8 groups.ab.
9 or/1-8
10 exp animals/ not humans.sh.
11 9 not 10

<u>Search terms in Psychinfo</u>
ADHD:
1 attention deficit disorder with hyperactivity.mp.
2 ADHD.mp. or exp Attention-Deficit-Hyperactivity Disorder/
3 hkd.mp.
4 exp Hyperkinesis/
5 hyperkine$.mp.
6 overactive.mp.
7 "ADHD".mp.
8 ADHD.tw.
9 ADDH.mp.
10 ("attention deficit" adj2 "hyperactivity").mp.
11 "ADHS".mp.
12 (("attention$" or "behav$") adj3 ("defic$" or "dysfunc$" or "disorder$")).mp.
13 ("impulsiv$" or "inattentiv$" or "inattention$").mp.
14 ("minimal" adj3 "brain" adj3 ("disorder$" or "dysfunct$" or "damage$")).mp.
15 ("hyperactiv$" or "hyper-activ$").mp.
16 exp Attention Deficit Disorder with Hyperactivity/
17 or/1-16

Psychostimulants:

1 (amphetamin$ or amfetamin$ or anfetamin$).mp.
2 benzedrin$.mp.
3 (dexamphetamin$ or dexamfetamin$ or dextroamphetamin$ or dextroamfetamin$ or dexedrin$).mp.
4 (dex-amphetamine or dex-amfetamine or dextro-amphetamine or dextro-amfetamine).mp.
5 (lis-dexamphetamin$ or lis-dexamfetamin$).mp.
6 exp Methylphenidate/
7 methylphenidate.mp.
8 OROS.mp.
9 Dexmethylphenidate.mp.
10 (Adderall or Amphetamine or Desoxyn* or Phenopromin or Amfetamine or Phenamine).ti,ab.
11 (Centramina or Fenamine or Levoamphetamine or Dexamfetamine or Dexamphetamine or Dexedrine).ti,ab.
12 (Dextroamphetamine or DextroStat or Oxydess or Methylamphetamine or Methylenedioxyamphetamine).ti,ab.
13 (Methamphetamine or Chloroamphetamine or Metamfetamine or Deoxyephedrine or Desoxyephedrine or Ecstasy).ti,ab.
14 (Concerta or Daytrana or Methylphenidate or Equasym).ti,ab.
15 (Methylin or Tsentedrin or Centedrin or Phenidylate or Ritalin$).ti,ab.
16 (Elvanse or Focalin or Dexmethylphenidate or Lisdexamfetamine or Vyvanse or Medikinet).ti,ab.
17 (Metadate or Quillivant).ti,ab.
18 exp Dextroamphetamine/
20 Lisdexamphetamine.mp.
21 Amphetamine.mp. or exp Amphetamine/
22 "Lisdex$".af.
23 "OROS".af.
24 "Mixed Amphetamine Salts".af.
25 "Mixed Amphetamine Salts".mp.
26 exp CNS Stimulating Drugs/
27 or/1-26

Other medications:
1 exp Clonidine/
2 exp Bupropion/
3 exp Pemoline/
4 exp Imipramine/
5 exp Desipramine/
6 (Atomoxetine or Biphentin or Bupropion or Amfebutamone or Zyntabac or Quomen or Wellbutrin or Zyban).ti,ab.
7 (Catapres* or Clonidine or Klofenil or Clofenil or Chlophazolin or Gemiton or Hemiton or Isoglaucon or Klofelin).ti,ab.
8 (Clopheline or Clofelin or Dixarit or Duraclon or Guanfacine or Estulic or Kapvay).ti,ab.

9 (Modafinil or Nexiclon or Strattera).ti,ab.
10 Atomoxetine.mp. or exp Atomoxetine/
11 Guanfacine.mp.
12 Modafinil.mp.
13 "Guanfacine".af.
14 "Modafinil".af.
15 "Clonidine".af.
16 "Bupropion".af.
17 or/1-16

Psychosocial interventons:
1 "Contingency Management".af.
2 "Management techniques".af.
3 "contingency techniques".af.
4 "psychosocial interventions".af.
5 "psychosocial treatment".af.
6 "psychosocial therapy".af.
7 "social skills training".af.
8 "social skills intervention".af.
9 "social skills treatment".af.
10 "problem solving intervention".af.
11 "problem solving treatment".af.
12 "problem solving training".af.
13 "problem solving therapy".af.
14 "behavior modification".af.
15 "cognitive behavior treatment".af.
16 "cognitive behavior therapy".af.
17 "cognitive behavior training".af.
18 "parent training".af.
19 "parent counseling".af.
20 "parent support".af.
21 "school-based".af.
22 "classroom-based".af.
23 "school intervention".af.
24 "classroom intervention".af.
25 "teacher training".af.
26 "after-school".af.
27 "remedial teaching".af.
28 "peer tutoring".af.
29 "computer assisted learning".af.
30 "task modification".af.
31 "curriculum modification".af.
32 "classroom management".af.

33 "education intervention".af.
34 "multimodal$".af.
35 "multimodal treatment".af.
36 "multimodal therapy".af.
37 "multimodal intervention".af.
38 "educational intervention".af.
39 "verbal self-instruction training".af.
40 "cognitive training".af.
41 "cognitive remediation".af.
42 "social skills training".af.
43 "social skills education".af.
44 behavior therapy.af.
45 or/1-44

Complementary interventions:
22 "few foods diet".af.
23 "elimination diet".af.
24 "oligoantigenic diet".af.
25 "restriction diet".af.
26 "food intolerance".af.
27 "food allergy".af.
28 "food hypersensitivity".af.
29 "food color".af.
30 "food dye".af.
31 "Feingold diet".af.
32 dietary supplements.mp. or exp Dietary Supplements/
33 exp Fatty Acids/ or omega-3.mp.
34 "Kaiser Permanente diet".af.
35 "K-P diet".af.
36 "tartrazine".af.
37 "azo dye".af.
38 "carmoisine".af.
39 "sunset yellow".af.
40 vitamin.mp. or exp Vitamins/
41 "St John's Wort".af.
42 "yoga".mp. or exp Yoga/
43 exp Mindfulness-Based Interventions/ or exp Mindfulness/ or mindfulness.mp.
44 alternative medicine.mp. or exp Alternative Medicine/
45 "valerian".af.
46 "tryptophan".af.
47 "melatonin".af.
48 exp Acupuncture/
49 "acupuncture".af.

50 " Tai Chi".af.
51 exp Osteopathic Medicine/ or osteopathic medicine.mp.
52 chiropractic.mp.
53 exp Osteopathic Medicine/ or osteopathic medicine.mp.
54 chiropractic.mp.
55 "cognitive training".af.
56 "attention training".af.
57 "working memory training".af.
58 "cognitive remediation".af.
59 "executive function training".af.
60 "neurofeedback".af.
61 "EEG biofeedback".af.
62 "neurotherapy".af.
63 "slow cortical potentials".af.
64 neurofeedback.mp. or exp Biofeedback, Psychology/ or exp Neurofeedback/ or exp Electric Stimulation Therapy/
65 or/1-64

Controlled trials:
1 random: assigned.tw.
2 "double-blind".tw.
3 control.tw.
4 or/1-3

Limiters:
Medline:
1 Search terms (e.g. ADHD AND Psychostimulants AND trial)
2 limit 1 to yr="2000 -Current"
3 limit 2 to "adolescent (13 to 18 years)"
4 adolescent*.ab. or adolescent*.ti.
5 2 and 4
6 3 or 5

Psychinfo:
1 Search terms (e.g. ADHD AND Psychostimulants AND trial)
2 limit 1 to yr="2000 -Current"
3 limit 2 to adolescence <13 to 17 years>
4 adolescent*.ab. or adolescent*.ti.
5 2 and 4
6 3 or 5

Table S3. Placebo-controlled studies of pharmacological treatment in adults with ADHD and comorbid SUD

Author, year	Population	Study design and treatment	Concurrent treatment	Treatment completers	Primary outcome measurement: ADHD	Primary outcome measurement: SUD	Primary outcome	Level of evidence (SIGN)
				Methylphenidate				
Schubiner, 2002[75]	48 adults aged 18-55 yrs (mean 37 yrs; 89.6% male) with ADHD and cocaine dependence	12-wk, RCT, DB, PC; parallel groups: MPH (max. 90 mg/day, titrated fixed dose) or placebo	12-wk group CBT for SUD and 12-wk individual CBT for ADHD	MPH: 45%; placebo: 58%	Mean change over time on self-reported ADHD Symptom Checklist by treatment group	Number of self-reported days of cocaine use past 30 days, and proportion of cocaine negative urine screens by treatment group	No significant difference between both groups in reduction of ADHD-symptoms (p=0.1098), self-reported cocaine use (p=0.354) and cocaine negative urine screens (p=0.512)	1 −
Carpentier, 2005[76]	25 adults (mean age 31.9 yrs; 88% male) with ADHD and SUD	8-wk, randomized, DB, PC, crossover study: 4x2-wk: MPH (max. 45 mg/day, fixed dose) or placebo	Ongoing inpatient, drug-free treatment	76% in both treatment conditions combined	Mean change over time on clinician-rated ADHD-RS-IV by treatment condition	NA (all patients abstinent during inpatient treatment)	No significant difference between both conditions in reduction of ADHD-symptoms (p=0.352)	2 −
Levin, 2007[77]	98 adults aged 18-60 yrs (mean 39.0 yrs; 57.1% male) with ADHD and opiate dependence, 53.1% of whom with co-occurring cocaine use disorder	12-wk, RCT, DB, PC; parallel groups: MPH-SR (max. 80 mg/day, titrated fixed dose) or bupropion (max. 400 mg/day, titrated fixed dose) or placebo	Methadone maintenance treatment; 12-wk individual CBT for SUD	MPH-SR: 65.6%; bupropion: 69.7%; placebo: 75.8%	% responders (≥ 30% reduction in ADHD-symptoms) on self-reported AARS, and % responders on both AARS and clinician-rated CGI (CGI-score ≤ 2 at endpoint) by treatment group	Proportion of weeks with any drug use, according to self-report and/or urine screens by treatment group	No significant difference between the three groups in % responders on AARS (p=0.48), AARS+CGI (p=0.42), and proportion of drug positive weeks (p=0.46)	1 −
Levin, 2007[78]	106 adults aged 18-60 yrs (mean 37 yrs; 83% male) with ADHD and cocaine dependence	14-wk RCT, DB, PC; parallel groups: MPH-SR (max. 60 mg/day, titrated fixed dose) or placebo	14-wk individual CBT for SUD	MPH-SR: 43.4%; placebo: 45.3%	% responders (≥ 30% reduction in ADHD-symptoms) on self-reported	Proportion of weeks with cocaine positive urine screens, and % patients with ≥ 2 weeks of continuous cocaine	No significant difference between both groups in % responders on AARS (p=0.44), proportion of cocaine positive weeks (p=0.69), and % patients	1 −

					AARS by treatment group	abstinence by treatment group	with prolonged cocaine abstinence (p=0.69)	
Konstenius, 2010[79]	24 adults aged 18-65 yrs (mean 37.4 yrs; 75% male) with ADHD and amphetamine dependence, with ≥ 4 weeks total abstinence prior to inclusion	12-wk, RCT, DB, PC; parallel groups: OROS-MPH (max. 72 mg/day, titrated fixed dose) or placebo	12-wk individual skills training for ADHD and SUD	OROS-MPH: 41%; placebo: 16%	Mean change over time on self- and observer-reported CAARS by treatment group	Proportion of negative urine drugs screens (amphetamine, other drugs) by treatment group	No significant difference between both groups in reduction of self-reported (p=0.137) and observer-reported (p=0.686) ADHD-symptoms and amphetamine (p=0.472) and other drug use (p=0.501)	1 −
Winhusen, 2010[80]	255 adults aged 18-55 yrs (mean 37.8 yrs; 56.5% male) with ADHD and smoking ≥ 10 cigarettes/day	11-wk, RCT, DB, PC; parallel groups: OROS-MPH (max. 72 mg/day, titrated fixed dose) or placebo	11-wk individual smoking cessation counseling, and 21 mg/day nicotine patch in weeks 4-11	OROS-MPH: 84.3%; placebo: 84.4%	Mean change over time on interviewer-rated ADHD-RS-IV and clinician-rated CGI by treatment group	% patients with prolonged, self-reported tobacco abstinence during weeks 7-11 (TLFB) by treatment group	Significant improvement in ADHD- symptoms on ADHD-RS-IV and CGI in OROS-MPH group vs placebo (d=0.62; p<0.0001); no treatment effect on tobacco abstinence (p=0.81)	1 +
Konstenius, 2014[81]	54 adults aged 18-65 yrs (mean 42 yrs; 100% male) with ADHD and amphetamine dependence, at the end of their prison term, with total abstinence from illicit drugs in the 2 weeks preceding study inclusion	24-wk, RCT, DB, PC; parallel groups: OROS-MPH (max. 180 mg/day, titrated fixed dose) or placebo	First 12 weeks: individual CBT for SUD. First 2 weeks: stay in prison. Last 22 weeks: outpatient, supervised, mandatory probation	OROS-MPH: 29.6%; placebo: 7.4%	Mean change over time on self-reported CAARS and clinician-rated CGI by treatment group	Proportion of negative urine drug screens (amphetamine, other drugs) by treatment group	Significant improvement in ADHD- symptoms on CAARS in OROS-MPH vs placebo (d=0.89; p=0.011); no effect on CGI (n.s); significantly more drug-negative urines in OROS-MPH group vs placebo (d=0.25; p=0.047)	1 −
Mixed amfetamine salts								
Levin, 2015[82]	126 adults aged 18-60 yrs (mean 40.4 yrs; 84.1% male) with ADHD and cocaine dependence	13-wk, RCT, DB, PC; parallel groups: MAS-XR (max. 60 or 80 mg/day, titrated fixed dose) or placebo	12-wk individual CBT for SUD, and progressive vouchers for	MAS-XR 80 mg: 79.1%; MAS-XR 60 mg: 75.0%;	% responders (≥ 30% reduction in ADHD-symptoms) on AISRS by treatment group	Odds of a cocaine negative week (urine screens and self-report) over time by treatment group	Significantly higher AISRS response rate in MAS XR 60 mg (d=0.91; p<0.001), but not in MAS-XR 80 mg (p=0.07) group vs placebo; significantly higher	1 +

			attendance at the clinic	placebo: 67.4%			cocaine abstinence in MAS-XR 80 mg (d=0.94; p<0.001) and MAS-XR 60 mg (d=0.59; p=0.02) group vs placebo	
Lisdexamfetamine								
Kollins, 2014[83]	32 adults aged 18-50 yrs (mean 31.4 yrs; 62.5% male) with ADHD and smoking ≥ 10 cigarettes/day	4-wk, RCT, DB, PC; parallel groups: LDX (max. 70 mg/day, titrated fixed dose) or placebo	Nicotine patch (max. 21 mg/day)	LDX: 82.4%; placebo: 93.3%	Mean change over time on clinician-rated CAARS by treatment group	% patients with ≥ 4 weeks of continuous nicotine abstinence (carbon monoxide level) by treatment group	Significant improvement in ADHD- symptoms in LDX group vs placebo (d=0.64; p=0.01); no treatment effect on tobacco abstinence (p=0.54)	1 +
Atomoxetine								
Wilens, 2008[84]	147 adults (mean age 34.6 yrs; 85% male) with ADHD and alcohol use disorder, with ≥ 4 alcohol abstinent days prior to randomization	12-wk, RCT, DB, PC; parallel groups: atomoxetine (max. 100 mg/day, titrated flexible dose) or placebo	No concurrent treatment for ADHD or SUD; 12-step participation allowed	Atomoxetine: 44.4%; placebo: 64.0%	Mean change over time on clinician-rated AISRS by treatment group	Time to relapse of self-reported heavy drinking (≥ 5 (males) or ≥ 4 (females) glasses/day, or ≥ 3 glasses/day continuously during ≥ 1 week; TLFB) by treatment group	Significant improvement in ADHD- symptoms in atomoxetine group vs placebo (d=0.67; p=0.007); no treatment effect on time to relapse of heavy drinking (p=0.93)	1 −
McRae-Clark, 2010[85]	38 of 78 (ITT) adults analyzed, aged 18-65 yrs (mean 29.4 yrs; 76.3% male) with ADHD and cannabis dependence	12-wk, RCT, DB, PC; parallel groups: atomoxetine (max. 100 mg/day, titrated flexible dose) or placebo	Individual MET for SUD in weeks 1-4	Atomoxetine: 47.4%; placebo: 36.8%	Mean change over time on clinician-rated WRAADS and clinician-rated CGI improvement scale by treatment group	Number of self-reported days of cannabis use (TLFB) during week 12 by treatment group	No significant difference between both groups in reduction of ADHD-symptoms on WRAADS (p=0.23); significant improvement in ADHD-symptoms on CGI (p=0.022) in atomoxetine group vs placebo; no treatment effect on cannabis use (p=0.44)	1 −

Note: ADHD = attention deficit/hyperactivity disorder; SUD = substance use disorder; RCT = randomized controlled trial; DB = double blind; SB = single blind; PC = placebo controlled; ITT = intention-to-treat; MPH = methylphenidate; OROS-MPH = osmotic-release oral system methylphenidate; MPH-SR = sustained release methylphenidate; LDX = lisdexamfetamine dimesylate; CBT = cognitive behavioral therapy; MET = motivational enhancement therapy; DSM-IV = Diagnostic and Statistical Manual of Mental Disorders; ADHD-RS-IV = DSM-IV ADHD Rating Scale; CAARS = Conners' Adult ADHD Rating Scale; CGI = Clinical Global Impression scale; AARS = Adult ADHD Rating Scale; WRAADS = Wender-Reimherr Adult Attention Deficit Disorder Scale; AISRS = ADHD Investigator Symptom Rating Scale; TLFB = Time Line Follow-Back calendar method; NA = not applicable.

Section and Topic	Item #	Checklist item	Location where item is reported
**TITLE**			
Title	1	Identify the report as a systematic review.	1
**ABSTRACT**			
Abstract	2	See the PRISMA 2020 for Abstracts checklist.	1
**INTRODUCTION**			
Rationale	3	Describe the rationale for the review in the context of existing knowledge.	1-2
Objectives	4	Provide an explicit statement of the objective(s) or question(s) the review addresses.	2
**METHODS**			
Eligibility criteria	5	Specify the inclusion and exclusion criteria for the review and how studies were grouped for the syntheses.	2-3
Information sources	6	Specify all databases, registers, websites, organisations, reference lists and other sources searched or consulted to identify studies. Specify the date when each source was last searched or consulted.	2-3
Search strategy	7	Present the full search strategies for all databases, registers and websites, including any filters and limits used.	2-3
Selection process	8	Specify the methods used to decide whether a study met the inclusion criteria of the review, including how many reviewers screened each record and each report retrieved, whether they worked independently, and if applicable, details of automation tools used in the process.	2-3
Data collection process	9	Specify the methods used to collect data from reports, including how many reviewers collected data from each report, whether they worked independently, any processes for obtaining or confirming data from study investigators, and if applicable, details of automation tools used in the process.	2-3
Data items	10a	List and define all outcomes for which data were sought. Specify whether all results that were compatible with each outcome domain in each study were sought (e.g. for all measures, time points, analyses), and if not, the methods used to decide which results to collect.	2-3
	10b	List and define all other variables for which data were sought (e.g. participant and intervention characteristics, funding sources). Describe any assumptions made about any missing or unclear information.	2-3

Section and Topic	Item #	Checklist item	Location where item is reported
Study risk of bias assessment	11	Specify the methods used to assess risk of bias in the included studies, including details of the tool(s) used, how many reviewers assessed each study and whether they worked independently, and if applicable, details of automation tools used in the process.	3
Effect measures	12	Specify for each outcome the effect measure(s) (e.g. risk ratio, mean difference) used in the synthesis or presentation of results.	3
Synthesis methods	13a	Describe the processes used to decide which studies were eligible for each synthesis (e.g. tabulating the study intervention characteristics and comparing against the planned groups for each synthesis (item #5)).	2-4
	13b	Describe any methods required to prepare the data for presentation or synthesis, such as handling of missing summary statistics, or data conversions.	3
	13c	Describe any methods used to tabulate or visually display results of individual studies and syntheses.	2-3
	13d	Describe any methods used to synthesize results and provide a rationale for the choice(s). If meta-analysis was performed, describe the model(s), method(s) to identify the presence and extent of statistical heterogeneity, and software package(s) used.	n/a
	13e	Describe any methods used to explore possible causes of heterogeneity among study results (e.g. subgroup analysis, meta-regression).	n/a
	13f	Describe any sensitivity analyses conducted to assess robustness of the synthesized results.	n/a
Reporting bias assessment	14	Describe any methods used to assess risk of bias due to missing results in a synthesis (arising from reporting biases).	3
Certainty assessment	15	Describe any methods used to assess certainty (or confidence) in the body of evidence for an outcome.	3
**RESULTS**			
Study selection	16a	Describe the results of the search and selection process, from the number of records identified in the search to the number of studies included in the review, ideally using a flow diagram.	4
	16b	Cite studies that might appear to meet the inclusion criteria, but which were excluded, and explain why they were excluded.	4
Study	17	Cite each included study and present its characteristics.	5-33

Section and Topic	Item #	Checklist item	Location where item is reported
characteristics			
Risk of bias in studies	18	Present assessments of risk of bias for each included study.	12 & 29
Results of individual studies	19	For all outcomes, present, for each study: (a) summary statistics for each group (where appropriate) and (b) an effect estimate and its precision (e.g. confidence/credible interval), ideally using structured tables or plots.	5-10; 16-27; 30-33
Results of syntheses	20a	For each synthesis, briefly summarise the characteristics and risk of bias among contributing studies.	
	20b	Present results of all statistical syntheses conducted. If meta-analysis was done, present for each the summary estimate and its precision (e.g. confidence/credible interval) and measures of statistical heterogeneity. If comparing groups, describe the direction of the effect.	n/a
	20c	Present results of all investigations of possible causes of heterogeneity among study results.	n/a
	20d	Present results of all sensitivity analyses conducted to assess the robustness of the synthesized results.	n/a
Reporting biases	21	Present assessments of risk of bias due to missing results (arising from reporting biases) for each synthesis assessed.	n/a
Certainty of evidence	22	Present assessments of certainty (or confidence) in the body of evidence for each outcome assessed.	n/a
**DISCUSSION**			
Discussion	23a	Provide a general interpretation of the results in the context of other evidence.	33-35
	23b	Discuss any limitations of the evidence included in the review.	33-35
	23c	Discuss any limitations of the review processes used.	34-35
	23d	Discuss implications of the results for practice, policy, and future research.	33-35
**OTHER INFORMATION**			
Registration and protocol	24a	Provide registration information for the review, including register name and registration number, or state that the review was not registered.	n/a
	24b	Indicate where the review protocol can be accessed, or state that a protocol was not prepared.	35

Section and Topic	Item #	Checklist item	Location where item is reported
	24c	Describe and explain any amendments to information provided at registration or in the protocol.	n/a
Support	25	Describe sources of financial or non-financial support for the review, and the role of the funders or sponsors in the review.	35
Competing interests	26	Declare any competing interests of review authors.	35
Availability of data, code and other materials	27	Report which of the following are publicly available and where they can be found: template data collection forms; data extracted from included studies; data used for all analyses; analytic code; any other materials used in the review.	35

From: Page MJ, McKenzie JE, Bossuyt PM, Boutron I, Hoffmann TC, Mulrow CD, et al. The PRISMA 2020 statement: an updated guideline for reporting systematic reviews. BMJ 2021;372:n71. doi: 10.1136/bmj.n71

Figure S1: PRISMA_2020_checklist

For more information, visit: http://www.prisma-statement.org/
